# DeBasher: a flow-based programming bash extension for the implementation of complex and interactive workflows with stateful processes

**DOI:** 10.1186/s12859-025-06108-1

**Published:** 2025-04-16

**Authors:** Daniel Ortiz-Martínez

**Affiliations:** Department of Mathematics and Computer Science, Universitat de Barcelona, Gran Via de les Corts Catalanes, 585, 08007 Barcelona, Spain

**Keywords:** Workflow, Pipeline, Workflow manager, Data flow programming, Flow-based programming, Bash, Workflow interactivity, Workflow triggers

## Abstract

**Background:**

Bioinformatics data analysis faces significant challenges. As data analysis often takes the form of pipelines or workflows, workflow managers (WfMs) have become essential. Data flow programming constitutes the preferred approach in WfMs, enabling parallel processes activated reactively based on input availability. While this paradigm typically follows a linear, acyclic progression, cyclic workflows are sometimes necessary in bioinformatics analyses. These cyclic workflows also present an opportunity to explore workflow interactivity, a feature not widely implemented in existing WfMs.

**Results:**

We propose DeBasher, a tool that adopts the flow-based programming (FBP) paradigm, in which the workflow components are in control of their life cycle and can store state information, allowing the execution of complex workflows that include cycles. DeBasher also incorporates a powerful model of interactivity, where the user can alter the behavior of a running workflow. Additionally, DeBasher allows the user to define triggers so as to initiate the execution of a complete workflow or a part of it. The ability to execute processes with state and in control of their life cycle also has applications in dynamic scheduling tasks. Furthermore, DeBasher presents a series of extra features, including the combination of multiple workflows at runtime through a feature we have called runtime piping, switching to static scheduling to increase scalability, or implementing processes in multiple languages. DeBasher has been successfully used to process 131.7 TB of genomic data by means of a variant calling pipeline.

**Conclusions:**

DeBasher is an FBP Bash extension that can be useful in a wide range of situations and in particular when implementing complex workflows, workflows with interactivity or triggers, or when a high scalability is required.

**Supplementary Information:**

The online version contains supplementary material available at 10.1186/s12859-025-06108-1.

## Background

In the rapidly evolving field of bioinformatics, the analysis of complex biological data presents significant challenges. As the volume and variety of such data continue to expand, the development of more adaptable, scalable, and efficient computational tools has become essential for effective data management and analysis.

Often, the analyses to be carried out take the form of pipelines or workflows, where a set of processes work coordinatedly to analyze the data. In these scenarios, the optimal utilization of computational resources is paramount, a goal that is closely related to the ability to exploit process parallelism.

Whenever advanced support for pipeline execution is required, workflow managers (WfMs) can be used. A WfM is software to automate the execution of various processes within a predefined sequence. The data flow programming paradigm has become the norm in bioinformatics WfMs as opposed to the traditional control flow approach. Data flow programming focuses on the movement of data through a series of computational components, where the execution of operations is driven by the availability of data. In this paradigm, components are activated as soon as their input data becomes available, leading to a model where process execution is inherently parallel and reactive.

In the standard data flow approach, execution is typically linear and can be effectively represented by a directed acyclic graph (DAG). In this model, each node in the graph represents a specific computational component, while the edges between nodes signify the flow of data from one component to the next. The acyclic nature of the graph ensures that data flows in a single direction, preventing any circular dependencies and allowing for a linear progression of tasks.

Many WfMs have been proposed so far, particularly in the field of bioinformatics [1]. Popular examples include Nextflow [2], Snakemake [3], Galaxy [4], Cromwell [5] or Toil [6], which have become essential for handling the increasingly complex data analyses required in modern bioinformatics. These tools follow the standard data flow paradigm mentioned above, ensuring a clear and structured way to manage computational tasks.

While the standard data flow paradigm is effective for many bioinformatics workflows, there are certain scenarios where the ability to execute cycles within a workflow would be required. These workflows may involve iterative processes, such as optimization loops, convergence checks, or repeated analyses that refine results based on intermediate outcomes. In these cases, the strict acyclic nature of traditional workflow managers can be a limitation, as they are designed to prevent any form of circular dependencies.

The execution of arbitrary cycles has been previously identified in [7] as an important workflow pattern that can arise in the context of workflow execution. It is also possible to find examples illustrating the necessity of executing cycles within the bioinformatics literature. For instance, in [8], an iterative workflow for assembling genomic datasets is provided. Another example is given in [9], where it is identified the necessity of executing an adaptive and iterative approach when sequencing viral genomes.

There have been efforts to incorporate the execution of cycles into the traditional data flow approach, further illustrating the validity of this workflow pattern. One interesting example would be the recurse experimental feature incorporated in Nextflow, which according to the existing documentation, allows to iterate the execution of a component or entire workflow a certain number of times, using the output generated in a particular iteration as the input of the next one. However, the proposed execution model would have severe constraints: i) the input and output type definitions should be identical for the component or workflow, ii) the number of cycles to be executed should be known in advance or depend on some condition of the output and iii) it is not clear how the components can store internal state information if that was necessary, due to the fact that at each iteration, the component or workflow is executed from the start, with the input as the only difference. The popular Snakemake WfM also incoporates a recursion feature that is non-experimental and more mature, but the proposed solution still keeps the third limitation mentioned above related to the ability to maintain internal state information. As far as we know, the most advanced tool that supports the execution of cycles would be Cylc [10]. This tool adapts the standard dataflow paradigm for its use in the execution of workflows with cycles. Although it is a very successful and advanced tool, it also simplifies the execution of workflows with cycles by not allowing processes to store state information.

The ability to execute workflows with cycles opens the door to exploring a feature that until now has not received much attention in previously existing WfMs: the possibility for the user to interact with a running workflow, altering its behavior. In a workflow based on the standard dataflow paradigm, the input parameters are known at the start of execution, making unnecessary any adjustment beyond re-executing the workflow. However, the situation changes when the workflow incorporates cycles. The aforementioned Cylc tool incorporates a feature that bears some relation to interaction, triggers. A trigger is an event defined by the user that initiates the execution of a workflow or a part of it. However, in this article we will consider that interaction exists when the workflow is running, and changes occur in its behavior that are caused by an external source. To the best of our knowledge, Cylc would not incorporate the possibility of interacting with a workflow.

One way to overcome the limitations of the standard data flow paradigm to execute complex workflows involving cycles would be to adopt a different data flow aproach called flow-based programming (FBP) [11]. In FBP, applications are defined as networks of interconnected components exchanging data, where each component functions as an independent module with well-defined inputs and outputs. FBP is able to work with linear workflows composed of components that are activated reactively, in the exact same way as the standard data flow approach does. However, it also incorporates a completely different working mode where the components run from the very beginning of program execution and are in control of their own life cycle. Such components communicate with others by sending information packets through connections, which are conceptually similar to UNIX pipes.

This ability of the processes to control their own lifecycle allows the execution of complex workflows that incorporate cycles. Additionally, the processes can include state information if necessary, further increasing the expressiveness that can be achieved.

Stateful processes that control their lifecycle can also have advantages in dynamic scheduling tasks. Dynamic scheduling is a fundamental part of many WfMs, as it allows the processes to be determined at runtime, increasing flexibility. However, since most WfMs adopt the standard data flow approach, the execution must evolve linearly, making certain tasks difficult, such as controlling the progression of a series of processes from another process. In contrast, a process that adopts the FBP approach could use this information to handle errors and retries, balance the workload or optimize performance measures.

To the best of our knowledge, there has been only one attempt to apply FBP to implement bioinformatic workflows: SciPipe [12]. SciPipe is a very interesting tool that allows the execution of workflows based on processes that run Bash commands (alternatively, the Go programming language can also be used). However, in SciPipe, the processes do not control their lifecycle; instead, they are executed reactively when they receive a complete set of input parameters. Additionally, it is not possible for the processes to store state information. Consequently, SciPipe implements a restricted model of FBP in which some of its most distinguishing features are absent.

In this article, we present DeBasher. A tool that fully adopts the FBP approach, allowing the execution of complex workflows, including workflows with cycles, where processes retain state information. The tool also allows this execution to be interactive or activated by means of user-defined triggers. As mentioned above, the fact that processes maintain state and are in control of their life cycle can also have advantages in dynamic scheduling tasks. Additionally, as will be explained in the following sections, DeBasher incorporates a series of extra features. Among these, we highlight the ability of combining multiple subworkflows at runtime without the need to recompile the entire workflow, an ability we have termed *runtime piping*. Another feature of DeBasher is the possibility to choose between dynamic and static scheduling as appropriate given the characteristics of the program to be executed. Choosing static scheduling can be useful to significantly increase scalability. On the other hand, DeBasher is language agnostic, which allows implementing the components that shape the programs through different programming languages.

DeBasher uses Bash as its native language. This decision has been strongly motivated by the fact that the tool is oriented towards the execution of UNIX-type commands, including both pre-existing commands and those implemented by the user through the tool, whether written in Bash or other languages using the language agnosticity feature. Finally, another motivation for using Bash as the native language lies in the good support it offers for one of the fundamental elements handled by DeBasher, UNIX FIFOs.

## Implementation

This section outlines the implementation of DeBasher and provides detailed guidance on how it can be utilized to develop programs. To illustrate its application, the section shows how to implement a DeBasher program and also references various code examples included in the supplementary materials. Several of these examples are centered around a specific program known as the Telegram problem, which will be discussed in the next section. Most of the DeBasher code listed in the supplementary materials is written in Bash because it is the tool’s native language, however, DeBasher is language agnostic.

### The Telegram problem

The Telegram problem is used in [11] to illustrate how FBP programs are designed. We explain it here because it is used within the article to illustrate concepts about DeBasher. More specifically, the Telegram problem involves creating a program that takes input lines of text and produces output lines with maximum word count, ensuring that each line’s character count remains under a specified limit. Words must remain intact without splitting, and it is assumed that no word exceeds the line’s length.

**Fig. 1:**
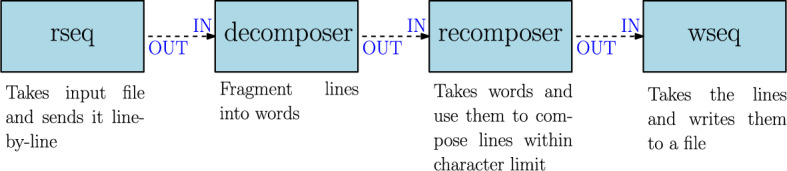
Diagram of the original implementation of the Telegram problem from an FBP perspective

The original implementation of the Telegram problem from an FBP perspective was proposed in [11]. In particular, the author mentions the necessity of defining four processes, which are depicted in Fig. 1. Those processes are the following:

- rseq: this process takes a file as input and reads it line by line, sending the file content to the decomposer process.
- decomposer: this process takes the lines of the input file and fragments them into words that are sent to the recomposer process.
- recomposer: the recomposer process takes as input the stream of words composing the input file provided by the decomposer process and the character limit per each line and generates a stream of text lines whose length is below the character limit. This stream is sent to the wseq process.
- wseq: finally, the wseq process takes the stream of text generated by the recomposer process and writes it to a file.

One important feature of the processes depicted in Fig. 1, is that they are executed concurrently. The reason why is because they use connections allowing to send data streams. Data stream communication is a hallmark of FBP, as will be explained in the next section.

### Flow-based Programming

DeBasher extends Bash by following the FBP paradigm. FBP combines two well known approaches:

- Dataflow programming: represents programs as directed graphs, showing data flow between operations. These operations are linked by defined inputs and outputs and execute as soon as all inputs are available.
- Component-based software engineering (CBSE): constructs software using modular, loosely-connected components. It prioritizes separation of concerns (SoC), a design principle that divides a program into sections, each handling a specific aspect of functionality. The primary goal of SoC is to enhance code modularity.

FBP structures a program as a network of independent processes. These processes communicate through connections, sending and receiving information packets (IPs) via named input and output ports. IPs, the basic units of data, carry the necessary data for computation along these connections.

In FBP, all processes start execution simultaneously and are in control of their life cycle. As a consequence of this, FBP processes can be stateful if needed, as opposed to the stateless steps executed in the standard dataflow approach. Alternatively, processes can be replaced by job steps if needed, which are executed reactively when their input data is available. While processes communicate by means of IPs, job steps use files, aligning more with a standard dataflow programming paradigm.

One key feature of FBP is that system design is split into two layers: the graph layer, which defines network configuration, and the component layer, which dictates data transformations. The component layer is typically textual, while the graph layer is visual, allowing FBP to naturally incorporate visual programming elements.

DeBasher allows to define FBP processes as commands receiving named options (equivalent to FBP ports). Due to the fact that DeBasher is language agnostic, such commands can be implemented in different languages. When the commands are implemented in Bash, Bash functions are defined. To implement commands in other languages, DeBasher uses *here documents* (HereDocs). Regarding the options, those starting with prefixes -out or --out define output ports, and the rest define input ports. To improve modularity, FBP processes can be grouped in modules implemented by means of Bash files.

A DeBasher program is a network of interconnected processes or job steps, configured by Bash functions or HereDocs. DeBasher uses UNIX named pipes (FIFOs) and regular UNIX files to communicate processes and job steps, respectively. Each module can define its program using processes and network configurations from itself or external modules, and can reuse programs from other modules.

One advantage of using pipes to communicate processes is that the processes can run simultaneously, increasing program efficiency. By contrast, the communication by regular files that characterize the standard dataflow paradigm necessarily results in the sequential execution of processes.

Another advantage of FBP is the ability to execute arbitrarily complex programs, including those having cycles. This would be a direct consequence of the above mentioned fact that FBP processes are in control of their lifecycle.

Finally, DeBasher itself is implemented using Bash and Python.

### Implementing a DeBasher program

In this section we will illustrate how to implement a DeBasher program, using the Telegram problem described in The Telegram problem as an example.

The first required step is to create the modules involved. Each module is associated to a file. In this case, we will only need one module, that will be stored in the debasher_telegram.sh file.

After the modules have been defined, we implement the processes intervening in the program. In our case, we will need to implement four processes called rseq, decomposer, recomposer and wseq. For each one, we should define a Bash function with the same name. The Bash function should read the input and output options at the start, and then incorporate the logic associated to the process.

For instance, the decomposer process should take as input the output of the rseq process, consisting in a text file sent line by line, fragment the lines into words and send the result to the output connection. Assuming that the input to the process is provided by means of the -inf option, and the output by means of the -outf option, the implementation of the decomposer process would be as follows:
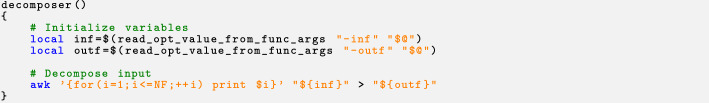


where the read_opt_value_from_func_args function is used to read the values for the options.

Once the four processes have been implemented, we can document those options for each process that should be provided by the user through the command line. Option documentation is later used by DeBasher to generate a help message for the program. In our program, the decomposer process does not receive any user option, but the rseq process receives the input file that should be processed. Assuming that the name of the option is -f, this can be achieved by means of the following function (the function name is obtained by concatenating the process name with the suffix _explain_cmdline_opts):
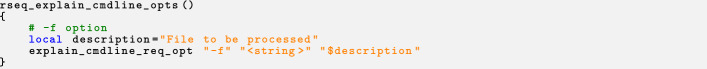


where the explain_cmdline_req_opt function defines the type of the value associated to the option as well as the option description.

After documenting the command line options, we can proceed to add the code that will specify the particular options that the process receives. This step, when completed for all of the processes, can be seen as defining the program network. For this purpose, we should specify how the inputs and outputs of each process are connected to the rest. Again, this is achieved by defining functions for the different processes. For the decomposer process, we should connect its input to the output of the rseq process. Also it should define a FIFO to send the output to the recomposer process. For this purpose, the following function is defined (the name of the function is composed by appending the suffix _define_opts to the name of the process):
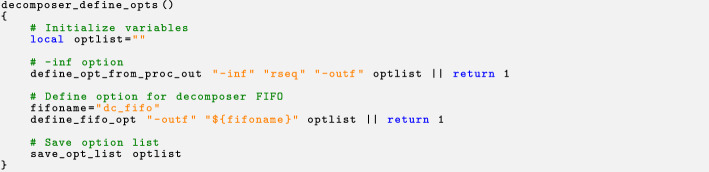


where the define_opt_from_proc_out function connects an option of the process being defined with an option of another process. The define_fifo_opt allows to define a FIFO and associate it to a process option. The options are added to the optlist variable. At the end of the function, the save_opt_list function is used to save the complete list of options for the process.

Finally, we define the program by adding all the processes that should participate. To achieve this, again we define a function (its name is obtained by concatenating the module name, debasher_telegram, with the suffix _program):
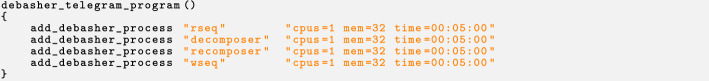


where the add_debasher_process function adds a process to the program. The function also allows to provide information about the computational and time requirements for each process.

It is worth highlighting that, due to the visual nature of FBP, the steps for documenting command-line options and defining the process interconnection network could be carried out through a graphical interface.

For additional implementation examples, Supplementary note 1 provides the Debasher version of “Hello World!”. Supplementary note 3 provides a Bash implementation of the above described Telegram problem and supplementary note 4 shows different ways in which such problem can be implemented with DeBasher. The complete implementation of the Telegram problem with four processes discussed above can be found in Supplementary note 4.1.

### Executing a DeBasher program

**Fig. 2:**
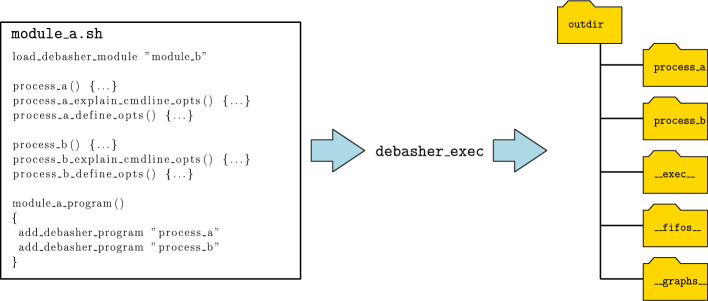
Execution of a generic DeBasher program using debasher_exec. As a result, a directory tree is generated

DeBasher incorporates specialized tools to work with programs. One of such tools is debasher_exec, that is used for program execution. Figure 2 depicts a generic program and the directory tree that is obtained after executing it by means of debasher_exec. More specifically, the program is composed of one module, module_a, and two processes, process_a and process_b. The module uses the add_debasher_module function to add or include another module, module_b. On the other hand, the directory tree generated includes output directories for both processes, process_a and process_b, a directory storing execution information, __exec__, a directory containing the process FIFOs defined (if any), __fifos__, and a directory storing graphs associated to the program, __graphs__. The generated graphs include process graphs, displaying how the process network is configured, or dependency graphs, illustrating how the execution of a particular process depends on the execution of other ones. Supplementary materials contain many examples of both kinds of graphs generated by DeBasher.

DeBasher incorporates other tools to work with programs. For instance, debasher_status can be used to figure out the execution status (pending, completed, failed, etc.) of the different processes that compose a given program. Another important tool would be debasher_stop, useful to stop a program being executed. Additional information about these tools and other ones can be found in the DeBasher home page.

### Exposure of process FIFOs

One attractive feature of DeBasher is its ability to handle user-defined triggers and interactivity, as it will be shown in User-defined triggers and interactive programs. The key element allowing the implementation of such feature is the exposure of process FIFOs to the user. Before a DeBasher program starts execution, FIFOs are created and stored in a particular folder of the output directory (see Fig. 2). This allows the user to easily interact with the program by sending to or receiving information from the FIFOs.

The exposed FIFOs can also be used to enable runtime piping, another DeBasher feature that will be explained in Runtime piping.

### Object-oriented programming

It has been noted that FBP and object-oriented programming (OOP) share parallels in key concepts like encapsulation and polymorphism [11]. DeBasher goes beyond those parallelisms introducing some OOP concepts of its own. Specifically, it fully characterizes program behavior by introducing two abstract classes, namely, the module and the process classes. User-defined modules and processes can be seen as subclasses and module- or process-related functions as methods. Methods are implemented as Bash functions or HereDocs (to enable language agnosticity), named by adding the method name as a suffix to the module or process name (HereDocs add an extra suffix to identify the language, e.g. py for Python). For example, in supplementary note 1, the module debasher_hello_world is defined. To implement its program method, we define the debasher_hello_world_program Bash function.

In order to satisfy program requirements, it is even possible to redefine particular methods. One fundamental example of this would be the redefinition of the define_opts method for processes, which constitutes one possible mechanism used by DeBasher to configure the program network, or in other words, to enable external definition of connections (see more about this below).

### External and distributed definition of connections

An essential element of FBP is the external definition of connections between processes [11], configuring the program’s network. These connections are defined outside the process implementation (e.g. in a main function), constituting an example of SoC and serving as a basic FBP reuse mechanism.

DeBasher goes beyond standard external definition of connections by allowing to define such connections in a distributed manner. Specifically, how a particular process is connected to the rest becomes a property of such process, further improving SoC and thus, code reuse. For this aim, DeBasher allows to define two alternative process methods: define_opts or generate_opts. One good example of how this strategy is useful for code reuse is the implementation of the Telegram problem with two processes shown in supplementary note 4.2. Two interesting variants of this program can be obtained by only changing the define_opts method of the processes (see supplementary notes 4.3 and 4.4).

Additionally, supplementary note 7 shows how the interconnection pattern of a program with two processes can be reconfigured by only redefining a method for one of them. Moreover, given a process, not only are its connections defined within a method of that process, but also whether the process executes loops or arrays. Supplementary note 6 shows an example of this.

### Imperative process execution

Dataflow process coordination may become too restrictive in certain situations. DeBasher allows to revert to purely imperative process execution when needed. Since processes are implemented as parameterized Bash functions, it is possible to call them within the code of another process, enabling the execution of specific control flow logic and, at the same time, maximizing code reuse and modularity. To illustrate this, an imperative implementation of the Telegram problem is given in supplementary note 4.5.

### Static and dynamic process scheduling

DeBasher provides two process scheduling modalities: static and dynamic. Both modalities have their advantages and disadvantages, and can be used depending on the requirements of the particular scenario. Static process scheduling is simpler since all the computations to be executed should be known beforehand. In contrast, with dynamic scheduling, the required computations can also be determined at runtime. Thus, dynamic scheduling is more flexible, but it is also more complex to implement and may require more computational work from the scheduler during program execution.

DeBasher’s static process scheduling strategy is based on early definition of process dependencies, and is more suited to execute job steps in a standard dataflow programming approach. The scheduling work can be carried out by a built-in scheduler incorporated in DeBasher. The built-in scheduler decides which processes can start execution or should wait at a given time. Assignment of computational resources in this context can be seen as an instance of the well known knapsack problem, for which efficient implementations are available.

Static process execution can also be carried out using external schedulers, such as Slurm^1^. This is particularly efficient, since under these circumstances, all processes are launched at the start of program execution and process coordination is achieved by specifying process dependencies that were resolved earlier. In this way, DeBasher utilizes the full capabilities of the external scheduler.

On the other hand, Debasher also implements dynamic process scheduling by means of its built-in scheduler. This scheduling modality would be appropriate for both job steps and FBP processes. Within each job step or FBP process, the built-in scheduler is able to execute an arbitrary number of computations, both locally or in external high performance computing (HPC) infrastructure.

## Results

Despite being designed to execute general-purpose programs, DeBasher is well-suited for executing workflows, including essential features such as: (1) parallel process execution with data dependency handling; (2) automatic error handling; (3) result reproducibility through containers or package managers; (4) generation of dependency graphs; and (5) compatibility with high performance clusters or cloud infrastructure.

However, DeBasher incorporates some non-standard characteristics that are described below. Additionally, in Sample bioinformatics workflow, we show an example of a bioinformatics workflow implemented with DeBasher that was applied over a very large dataset.

### Language agnosticism

Taking advantage of the fact that DeBasher is language agnostic, it is easy to implement the “Hello World!” program in other languages. Currently, DeBasher provides support for four languages: Python, R, Perl and Groovy. Figure S3 shows the required code. Moreover, it would be straightforward to extend DeBasher to any other language.

One interesting advantage of the language agnostic capabilities of DeBasher lies in the fact that they are based on HereDocs. In particular, the use of HereDocs allows to define code in any language without the necessity of escaping any characters or making any other modification.

### Data streaming

The ability to handle data streams is a foundational aspect of FBP, and DeBasher implements it using UNIX FIFOs. There are many examples in the supplementary materials illustrating how data streams are used in DeBasher programs. One example would be the implementation of the above mentioned Telegram problem (see supplementary note 3).

### Arbitrary cycles with stateful processes

**Fig. 3:**
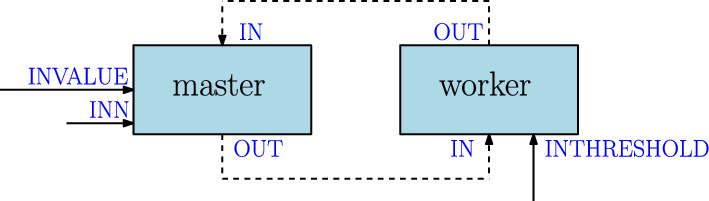
Example of a program with cycles, simulating a master-worker process organization

DeBasher incorporates the ability to execute programs with arbitrary cycles, due to the fact that FBP allows to define processes that are in control of their lifecycle. Moreover, such processes can be stateful. Figure 3 shows one example of a program where two processes, master and worker are cyclically connected. The program simulates a *master-worker* situation, where master requests worker to carry out computations, with the peculiarity that worker maintains state information. This state information will determine the type of computation that will be carried out.

More specifically, master uses a pipeline to send a numeric value to worker (involving port OUT for master and port IN for worker), and worker transforms the value, accumulates the result in an internal variable, and uses another pipeline to return the transformed value to master (involving port OUT for worker and port IN for master). The computation carried out by worker is very simple, it will add one to the value if the current cummulative value is below or equal to a threshold given by the user (INTHRESHOLD port), or two otherwise. The cycle is repeated until master receives a value greater than another one given as input parameter (INN port). At the beginning of execution, master also receives the initial number for doing the calculations (INVALUE port). Before terminating, worker prints the cummulative value to the standard output. Figure S32 shows the code of the processes.

### Dynamic scheduling with stateful processes

**Fig. 4:**
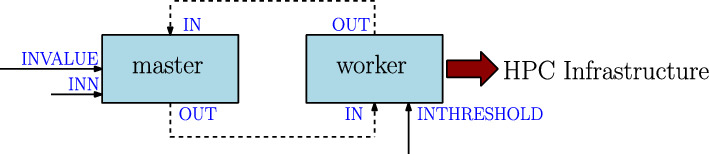
Example of a program with cycles performing dynamic scheduling

DeBasher is able to allocate computations dynamically. Starting from the program with cycles explained in the previous section, the computations carried out by the worker process can be executed taking advantage of the specific support provided by DeBasher. DeBasher can, for instance, handle the execution of the computation by means of HPC infrastructure. Figure 4 shows a graphical example of this, where the worker process replaces the previous local computations by HPC infrastructure computations. The code that is required to implement the example is shown in Figure S33. Again, one interesting feature of the implemented code is that the processes can be stateful, allowing to implement arbitrarily complex logic.

### User-defined triggers and interactive programs

Until this point, connections have been used to communicate two processes. However, with DeBasher, connections can be used to link a process to the outside for input or output. This is possible because DeBasher exposes the FIFOs intervening in a given program, as it was explained in Exposure of process FIFOs. Communicating a process to the outside has two interesting utilities: user-defined triggers and user interaction with a program.

User-defined triggers allows the user to define events that produce an action or a set of actions. One example could be an event that results in the execution of a whole workflow because new data to be processed has become available. Another example could be an event used to execute a workflow periodically. Going back to the master-worker example, the INVALUE option defining the initial value used by the master process could be replaced by a FIFO connecting the process with the output of a command scheduled periodically by means of the UNIX cron daemon.

On the other hand, user interaction is enabled when the user can alter the behavior of a program being executed. Using again the previous master-worker example, one interaction example would be the ability to redefine the input threshold for the worker, so that the user can provide specific values. For this purpose, the INTHRESHOLD port would be connected to the outside by means of a FIFO.

**Fig. 5:**
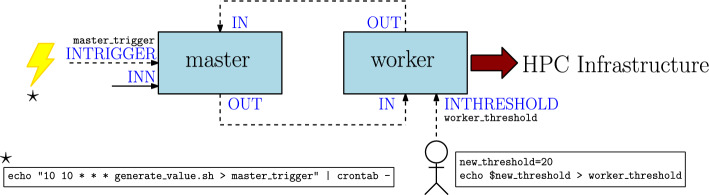
Example of a program incorporating a trigger and interactivity

Figure 5 shows a diagram for the master-worker example introducing triggers and interactivity. The INVALUE port has been renamed to INTRIGGER and receives as input the output of a script called generate_value.sh periodically executed by means of cron (we assume that the name of the FIFO is master_trigger). On the other hand, the INTHRESHOLD port for the worker now allows the user to modify the input threshold at each execution, without the necessity of restarting the program (it is assumed that the corresponding FIFO is called worker_threshold). Figure S35 shows the new code for the processes.

### Runtime piping

There is one additional utility of connecting a process to the outside using FIFOs: a mechanism to combine DeBasher programs that we will call *runtime piping*. Runtime piping can be seen as the equivalent in a workflow execution scenario to the concept of dynamic linking in a conventional program execution scenario. In particular, runtime piping allows the user to connect two or more workflows currently being executed. This would constitute a step beyond in terms of extensibility, since there is no need to rebuild an entire workflow when a particular component or subworkflow is changed.

**Fig. 6:**
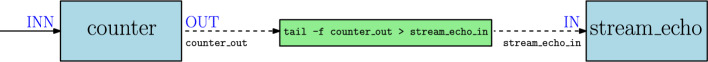
Example of connecting the output of a program to the input of another one

The simplest example of runtime piping would be connecting the output of a program to the input of another one. Let us suppose we have a simple program with one process, counter, that receives a number as input (port INN) and generates a count from zero to that number as output (port OUT). Additionally, we have another program with one process, stream_echo, that just receives a stream as input (port IN) and prints it to the standard output. Figure 6 shows how the two programs can be connected with a simple tail command assuming that the output FIFO of the counter process is called counter_out and the input FIFO of the stream_echo process is called stream_echo_in. The code of both processes is discussed in supplementary note 11.

**Fig. 7:**
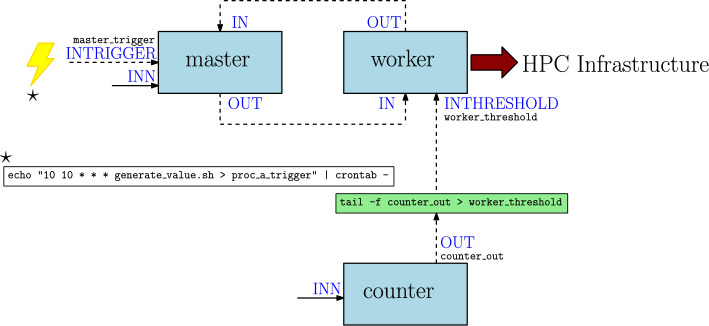
Example of using the output of a program to alter the behavior of another one

It is worthy of note that runtime piping is not limited to connecting programs serially. The output of a program could be connected to a particular module of another one, altering its behavior. Figure 7 shows an example of this, where the counter process is used to provide the threshold received by the worker process in the above described master-worker example.

### Choosing static scheduling for increased scalability

Dynamic scheduling provides the highest flexibility when implementing and executing workflows. However, there are situations where such flexibility is not required and resorting to static scheduling can constitute a valuable option to reduce scheduling overhead and increase predictability of resource usage (due to the fact that all processes to be scheduled are known in advance). This results in increased scalability with respect to dynamic scheduling.

To test the advantages of the static scheduling modality implemented in DeBasher, we decided to adopt the same methodology used in [13] to study scalability, where the performance of a set of WfMs is evaluated when executing an increasing number of small processes. It is important to highlight that, although the proposed experimentation setting constitutes an idealized execution scenario, it is still closely related to real use cases when executing bioinformatics workflows. This is discussed in more detail in Discussion.

More specifically, we compared DeBasher with three workflow languages incorporating dynamic scheduling: Nextflow, Common Workflow Language^2^ (CWL) and Workflow Description Language^3^ (WDL), in line with the experimentation carried out in [13]. The comparison was made by executing an increasing number of instances of a one-step program (host_process) or a two-step program (host_workflow). The host_process program runs a single process named host1, which executes the hostname UNIX command. The host_workflow program runs both host1 and host2 processes, with host1 running first, followed by host2, which also executes the hostname command. We refer to a single execution of process host1 or host2 as a *workflow task*. The experiments were executed in an AWS Slurm ParallelCluster with 1 head node and 32 computing nodes. Each node had 2 CPUs and 4GB of RAM. We used Toil (v7.0.0) as execution engine for CWL, and Cromwell (v87) for WDL. Nextflow (v24.04) comes with its own engine.

**Fig. 8:**
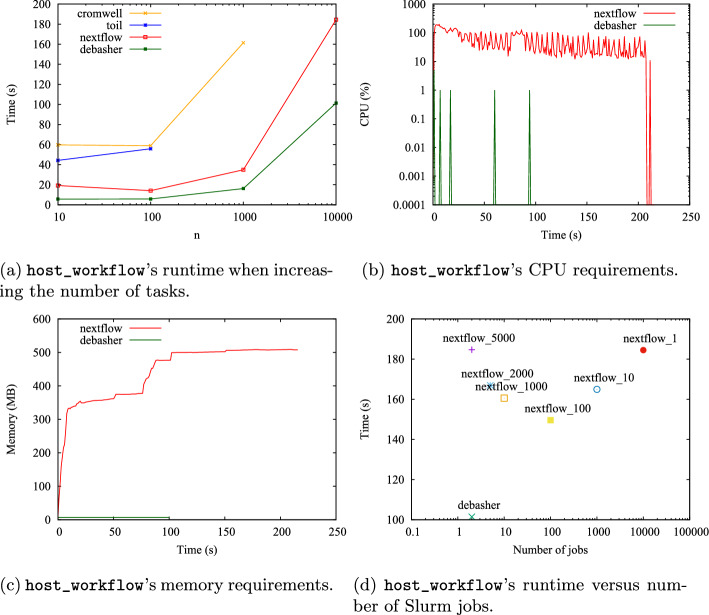
Scalability experiments for CWL, WDL, Nextflow and DeBasher

Figure 8a shows how the runtime of host_workflow evolved as the number of workflow tasks increased, from 10 to 10K. Cromwell and Toil could not finish execution for 1K and 10K tasks, respectively. Both tools were significantly slower than DeBasher. Nextflow was able to complete execution in all cases but was slower than DeBasher. Notably, when executing 10K tasks, DeBasher achieved a 45% runtime reduction with respect to Nextflow. Moreover, it should be noted that Nextflow needed to submit one Slurm job per task, causing important overhead. Figures 8b and  8c measured the host_workflow’s CPU and memory requirements, respectively, for DeBasher and Nextflow when executing 10K tasks. As it can be seen, Nextflow consumed substantial resources during the whole workflow execution (around 1 CPU and 500MB of RAM), while DeBasher requirements where virtually zero.

DeBasher outperforms Nextflow due to its static process scheduling approach. It resolves process dependencies at the start and submits all necessary Slurm jobs immediately. In the experiments, only two jobs were needed (one for host1 and one for host2) because DeBasher can launch Slurm arrays. Interestingly, version 24 of Nextflow now supports Slurm arrays, allowing to fragment the execution of a set of tasks in fixed-size arrays. Figure 8d shows the runtime for executing host_workflow with 10K tasks based on the number of submitted Slurm array jobs. DeBasher required only two arrays and was in all cases faster than Nextflow, whose array count depended on the array size. Curiously, Nextflow’s execution time decreased starting from array size 1 until 100, but increased for higher ones, forcing the user to make a trade-off between runtime and number of submitted jobs. For unclear reasons, higher array sizes made task execution slower. This explains why increasing the array size reduces runtime only up to a point: Nextflow can submit more tasks at once, but the computational cost for each task rises.

When using Nextflow arrays, and due to the fact that array execution is managed by the Slurm scheduler, it is anticipated that Nextflow’s CPU and memory requirements will decrease as the array size increases. This is because fewer jobs need to be submitted to the scheduler. However, this may not be the case, since Nextflow does not follow a static scheduling approach, but a dynamic one, where the majority of the scheduling work is carried out by Nextflow itself. We executed new experiments to clarify this.

**Fig. 9:**
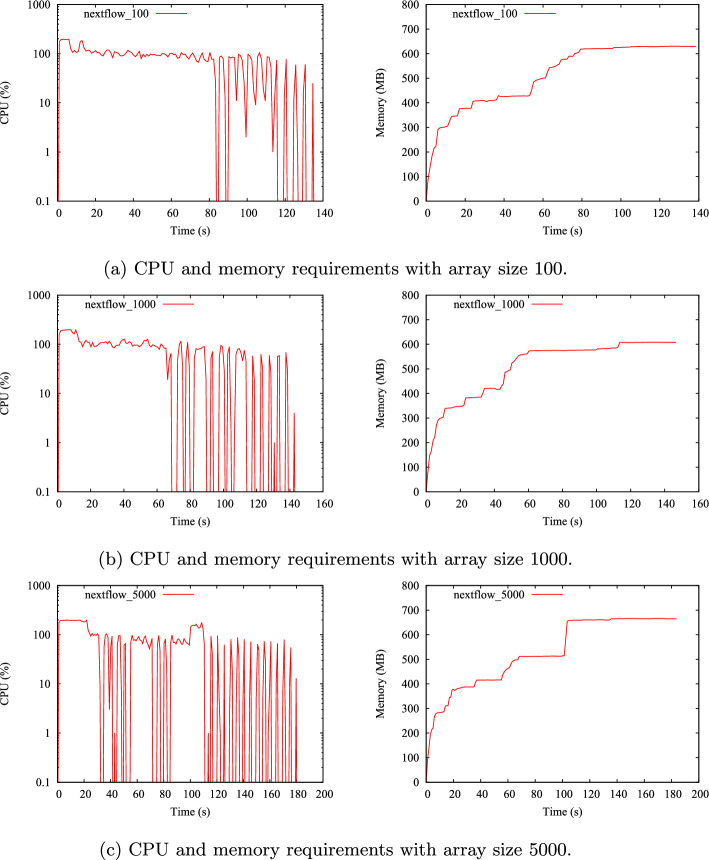
Evolution of Nextflow’s CPU and memory requirements when executing the host_workflow program with 10K tasks for different values of the array size

Figure 9 shows the evolution of Nextflow’s CPU and memory requirements when executing the host_workflow program with 10K tasks using arrays of size 100, 1 000 and 5 000. As it can be seen, Nextflow required a non-negligible amount of RAM (up to 600MB) no matter the size of the array. Regarding the CPU usage, it is observed for all cases a 100% CPU consumption for a certain time period from the very beginning of the execution, followed by an intermittent CPU consumption pattern. This suggests that Nextflow is active during the whole program execution, even when the number of arrays to be executed is small. This is particularly interesting for the experiment with an array size equal to 5 000, where only 2 Slurm arrays needed to be executed.

Furthermore, examining the overhead incurred by Nextflow and DeBasher in managing process dependencies is of particular interest. For this purpose, we compared the time cost of executing the same number of workflow tasks for host_process and host_workflow. Results show that DeBasher introduced virtually no overhead, whereas that of Nextflow was noticeable (see supplementary note 12.2.1).

Finally, the ability of Nextflow and DeBasher to distribute tasks across cluster nodes was assessed by analyzing the output of the hostname command. The deviation from the ideal distribution (equal processes per node) was quantified. Results indicate that DeBasher outperformed Nextflow for all the array sizes tested (see supplementary note 12.2.2).

### Sample bioinformatics workflow

To conclude this section, we show a sample bioinformatics workflow for variant calling implemented with DeBasher. Figure S43 shows a process graph incorporating all the processes involved in the implemented workflow, including their input and output options, as well as how the processes are connected. Additionally, Figure S44 shows a dependency graph, determining when each process can start execution. Both graphs were automatically generated with DeBasher.

The bioinformatics workflow that was implemented is useful to analyze genome sequencing normal-tumor pairs using a wide range of tools, automating the whole process, including sample downloading. Below we show a list of the variant calling tools that were executed within the workflow:

- Manta: a method to discover structural variants and indels from next generation sequencing data [14].
- Strelka2: an open-source small-variant-calling method for research and clinical germline and somatic sequencing applications [15].
- FACETS: a tool to analyze allele-specific copy number analysis from next generation sequencing (NGS) data [16].
- CNVkit: a tool for genome-wide copy number detection and visualization from targeted DNA sequencing [17].
- LUMPY: a probabilistic framework for structural variant discovery [18].
- SVTyper: performs breakpoint genotyping of structural variants (SVs) using whole genome sequencing data [19].
- DELLY: a structural variant discovery method that integrates short insert paired-ends, long-range mate-pairs and split-read alignments to accurately delineate genomic rearrangements at single-nucleotide resolution [20].
- MSIsensor-pro: a multinomial distribution model to quantify polymerase slippages for each tumor sample and a discriminative site selection method to enable microsatellite instability detection without matched normal samples [21].

The bioinformatics workflow described above was run over two whole genome sequencing cancer datasets: the melanoma dataset MELA-AU (dataset ID: EGAD00001003388; 183 individuals) and the esophagus dataset ESAD-UK (dataset ID: EGAD00001003580; 303 individuals). Both datasets are available at https://ega-archive.org/. The MELA-AU and ESAD-UK datasets contain 54.5 TB and 77.2 TB of data, respectively, resulting in a total of 131.7 TB of data that were successfully analyzed using DeBasher by means of the tools mentioned above.

For workflow execution, an HPC system consisting of three nodes was used. One node was equipped with two Intel Xeon E5-2697 CPUs, while the other two featured two Intel Xeon Gold 6148 CPUs each. The first node had 256 GB of RAM, whereas the second and third nodes each had 384 GB. The implemented pipeline utilized DeBasher’s static scheduling functionality, as dynamic scheduling was not required.

From the whole set of results obtained by DeBasher for MELA-AU and ESAD-UK, those generated with Strelka2 and FACETS were used to write a scientific study [22].

Computational cost measures of the workflow are not available, due to the fact that its execution was focused on generating results reported in [22], rather than measuring the efficiency of DeBasher. Other difficulties also played a role, including the fact that the HPC system was not exclusively available for the experiment, or that the storage space available on the HPC system only allowed a limited number of samples to be processed simultaneously due to their large size (over 100 GB each). Nevertheless, the execution of the workflow proved to be extremely robust, with errors occurring only with a few isolated samples, and it was possible to automate the entire analysis process, including the downloading of the samples.

## Discussion

In this section we discuss the previously shown results and compare the main features of DeBasher with those of well established tools.

Table 1 shows a feature comparison for DeBasher and a set of well known WfMs, including CWL, WDL, Nextflow, Snakemake, Scipipe and Cylc.

**Table 1:** Comparison of WfMs

	CWL$^1$	WDL$^2$	Nextflow	Snakemake	Scipipe	Cylc	DeBasher
Platform	CWL	WDL	Groovy	Python	Go	Cylc	Bash
Built-in multilanguage$^3$	Bash	Bash	Any	Bash	Bash	Bash	Any
CWL$^4$	Yes	NA	No	No	No	No	No
Workflow modules	Yes	Yes	Yes	Yes	Yes	Yes	Yes
GUI	Yes	Yes	Yes	Yes	No	Yes	No
Graph rendering	Yes	Yes	Yes	Yes	Yes	Yes	Yes
Reproducibility	Yes	Yes	Yes	Yes	No	No	Yes
Batch schedulers	Yes	Yes	Yes	Yes	Restricted$^5$	Yes	Yes
Distributed clusters	Yes	Yes	Yes	Yes	No	Yes	No
Cloud	Yes	Yes	Yes	Yes	No	Yes	Yes
Data streams	Yes	No	No	Yes	Yes	No	Yes
Stateful processes	No	No	No	No	No	No	Yes
Static scheduling	No	No	No	No	No	No	Yes
Dynamic scheduling	Stateless	Stateless	Stateless	Stateless	Stateless	Stateless	Stateful
Support for cycles	No	No	Exper$^6$	Stateless	Stateless	Stateless	Stateful
User-defined triggers$^7$	No	No	No	No	No	Stateless	Stateful
Interactive workflows	No	No	No	No	No	No	Yes
Runtime piping	No	No	No	No	No	No	Yes

$^1$CWL implemented with Toil

$^2$WDL implemented with Cromwell

$^3$Built-in support to execute code in multiple languages, meaning that the workflow language provides built-in syntax and features that facilitate the direct execution of tasks in different languages, making it more seamless and transparent. This contrasts with more basic integration mechanisms, where manual invocation (e.g., calling an external script or using shell commands) is required

$^4$Support for Common Workflow Language

$^5$According to the documentation, the feature is under development

$^6$This feature is currently classified as experimental in the tool’s documentation

$^7$The ability of the system to work with triggers arbitrarily defined by the user (more standard triggers, such as workflow or step reexecution due to changes in source code or input parameters, that require the user to explicitly invoke the WfM are not considered here)

DeBasher incorporates three distinctive features that are uncommon in other WfMs. First, it adopts a well-developed interactive execution model for workflows. This capability has been previously identified as increasingly important in the development of WfMs [23]. Second, DeBasher also offers a feature that we have called runtime piping (see Runtime piping). Runtime piping allows for arbitrarily connecting different workflows at runtime, favoring code extensibility. In conventional WfMs, this can only be done by recompiling all the code through subworkflows. Third, DeBasher is the only tool among those included in the feature comparison that allows working with stateful processes.

Workflow interactivity has received little attention when implementing WfMs in the field of bioinformatics, as it can be observed in Table 1. Outside this context, one WfM example incorporating interactivity would be the Jupyter-workflow (Jw) system [24], where Python notebooks are adapted for workflow definition and execution, using the code cell as the unit of computation, and maintaining the ability of interactively executing code. However, this approach would drastically differ from the one adopted in DeBasher, since Jw only allows interactive execution in a sequential manner, that is, code cells should be executed one by one, reducing the ability of the system to exploit parallelism. In contrast, DeBasher does not make this assumption, enabling interactivity at an arbitrary number of workflow sections simultaneously, without necessarilly stopping the concurrent execution of other ones.

On the other hand, the ability of DeBasher to work with stateful processes is especially appropriate for implementing complex workflows, in particular those containing cycles, as in this situation, maintaining information about previous iterations when executing a new one has important advantages. Specifically, state information can be useful for purposes such as managing dependencies between iterations, controlling the cycle termination condition, error handling or managing intermediate data. Among the tools analyzed, Nextflow only provides experimental support for cycles. Such support is more developed in Snakemake, but none of these tools can handle stateful processes. SciPipe also theoretically allows cycles with stateless processes, but its documentation does not mention anything about it. Finally, although not listed in Table 1, CWL has been contributed an extension that supports cycles [25]. Such an extension is not part of the CWL v1.2 standard, but apparently there are plans to incorporate it for CWL v1.3.^4^

The only tool among those included in the feature comparison that provides advanced support for cycles would be Cylc. Despite this, Cylc does not allow processes to have state. This leads to a simpler execution model but introduces significant limitations. Stateless processes are forced to resort to certain techniques to achieve results similar to those obtained through stateful processes, decreasing their expressiveness and efficiency. A good example of this would be the need to incorporate state information in the form of input parameters for the processes. Typically, the maintenance of such state information will require the use of external storage resources. This problem is particularly challenging in those scenarios where a shared filesystem or direct I/O between compute nodes is not available. Under these circumstances, state maintenance may require advanced external systems such as distributed databases or message queues, resulting in increased latency, higher network bandwidth usage, and the need for serialization and deserialization. Scalability can also become problematic due to bottlenecks in external storage systems and challenges in partitioning state across nodes. Fault tolerance and consistency are also harder to achieve, as maintaining durable and synchronized state across distributed processes is complex without shared resources. Finally, code maintenance and debugging under the circumstances discussed is also more difficult, as the state is external and dynamic, requiring careful monitoring.

In contrast, DeBasher’s FBP processes, being in control of their life cycle and therefore capable of maintaining state information, would not have any of these limitations.

In addition to the WfMs analyzed in Table 1, there are other approaches for incorporating cycles and stateful processes during workflow execution. Although these approaches would be less popular in the bioinformatics field, they are worth mentioning. The Autosubmit WfM [26] allows to repeat multiple times a stateless job. ecFlow^5^ is a WfM used at the European Centre for Medium-Range Weather Forecast (ECMWF) that defines workflows as a collection of stateless tasks that can be repeated. dispel4py [27] is a Python WfM that has been recently extended to support stateful computations [28]. However, dispel4py has a limited ability to handle cycles, due to the fact that it works with workflows in the form of DAGs. As a result, process state is primarily relevant only when the same workflow is executed multiple times with different inputs. Additionally, in the distributed and concurrent systems literature, stateful processes have been incorporated into the so-called *actor model*, where the fundamental unit of computation is the actor. In this model, an actor encapsulates state and behavior, interacting with other actors through message passing. One example of an actor model framework supporting stateful processes would be Ray [29], focused on artificial intelligence applications. However, the actor model is a control-driven framework, as opposed to the data-driven approaches that have become popular in the field of bioinformatics. In the actor model, data flow is not explicitly defined, instead it emerges from message-passing interactions between actors. Also the concurrency is no longer managed implicitly through data availability. These factors, stemming from the adoption of a control-driven approach, may make the actor model feel less natural when implementing bioinformatics pipelines.

DeBasher also incorporates a powerful model to handle user-defined triggers. To the best of our knowledge, from the tools being analyzed, Cylc would be the only tool that explicitly incorporates triggers into its execution model. However, Cylc triggers would be more restricted than those of DeBasher, due to the inability to keep process state.

An additional noteworthy feature of DeBasher is its language-agnostic nature, providing built-in support for the execution of tasks written in multiple programming languages. No matter whether the code is written in Bash or in other languages, it is treated as the implementation of a process that is automatically incorporated into the abstraction provided by DeBasher. As a result, users can incorporate diverse code directly into the workflow with no extra effort. From the other WfMs studied, only Nextflow incorporates built-in support for multiple languages (by means of its so-called script section). However, DeBasher would present an advantage over Nextflow in this area. In particular, since the feature is based on Bash HereDocs, it is not necessary to use escaped characters in the code, making the implementation process easier and more natural.

On the other hand, DeBasher is strongly based on the use of data streams to provide its distinctive features. However, due to the usefulness of data streams in various scenarios, there are other tools that already incorporate this feature, such as CWL implemented through Toil, Snakemake, or SciPipe.

DeBasher implements dynamic scheduling techniques, as do the rest of the analyzed tools. However, the use of stateful processes that are in control of their lifetime can provide advantages when a particular job should schedule other jobs. Such advantages include tracking the execution of tasks in real time, tracking available resources and using this information for load balancing, executing long-running or event-driven workflows where the decisions should be made incrementally, improved handling of job failures and retries, adaptive scheduling based on performance metrics, more flexible and customizable scheduling policies, etc.

Additionally, DeBasher is the only tool that allows adopting a static scheduling approach. In situations where the workflow to be executed is composed of tasks that are predictable, independent and uniform in resource requirements, the alternative of static scheduling can be interesting due to the low resource consumption and high scalability it allows to achieve, particularly when combined with external schedulers such as SLURM. The low resource consumption can be an advantage because jobs usually have to be launched from a head node, which typically has limited capacity. In this scenario, DeBasher’s static scheduling would launch all processes from the start, requiring hardly any subsequent intervention, allowing better resource allocation by SLURM and without CPU and memory requirements on the head node affecting other users. These advantages have been demonstrated in the scalability experiment reported in Choosing static scheduling for increased scalability, in which DeBasher was the only tool along with Nextflow that allowed executing a workflow composed of 10K tasks, with DeBasher also presenting a virtually zero consumption of computational resources both in CPU and memory.

It is important to highlight here that, contrary to what it may seem, the scalability experiment performed represents a highly valid use case of a WfM within a bioinformatics analysis context. WfMs are not only used to execute a few very long processes, but may also be necessary to execute a large number of small processes. In fact, the existence of this use case was what motivated the introduction of SLURM arrays in Nextflow. This can be verified by reading the discussion associated with the feature in the tool’s repository.^6^ Additionally, the use case considered aligns closely with the so-called embarrassingly parallel problems, a broad category of problems where the static scheduling capability of a WfM can be advantageous compared to dynamic scheduling. Practical examples of such problems, which have been addressed using distributed computing infrastructure, include the Folding@home [30], SETI@home [31], or Rosetta@home [32] projects, as well as Monte Carlo simulations, which are widely used in fields like finance, physics, and engineering.

DeBasher currently presents two main limitations. First, it lacks a graphical interface unlike the majority of the tools analyzed. However, this would be a temporary limitation, since, as explained in Flow-based Programming, the FBP paradigm is visual by nature, and thanks to the fact that the code defining the interconnection pattern of processes is completely separated from the rest of the code (see External and distributed definition of connections), it should be very easy to incorporate such an interface. Second, DeBasher incorporates a limited capacity for interoperation with HPC infrastructures, not allowing tasks to be executed in distributed systems. However, extending this ability should not be difficult based on the current capabilities of the tool, constituting one of the main lines of future work that are contemplated along with the development of the graphical interface mentioned above.

## Conclusions

DeBasher adopts the FBP paradigm to enable the implementation of complex workflows that can incorporate cycles. In contrast to other tools, the processes that compose a given workflow can retain state information, resulting in increased expresiveness. The greater ability to implement workflows with cycles opens the possibility of exploring a WfM feature that have previously received little attention: workflow interactivity. DeBasher leverages process connections implemented as UNIX FIFOs to provide a powerful interactivity model where the user can alter the behavior of a workflow at runtime. UNIX FIFOs can also be used to implement user-defined triggers, that cause the execution of a workflow or a part of it. Additionally, DeBasher offers enhanced extensibility through its ability to combine multiple workflows being executed. We refer to this ability as runtime piping. FBP processes, being in control of their life cycle, are also potentially useful for dynamic scheduling tasks. On the other hand, DeBasher offers interesting features not related to the execution of complex workflows, such as the ability to adopt a static scheduling approach for increased scalability or the capability to implement processes in any programming language, due to the fact that DeBasher is language agnostic. Finally, DeBasher has been sucessfully used to process 131.7 TB of genomic data by means of a variant calling pipeline.

## Additional file

Supplementary Material 1.

## Data Availability

Project name: DeBasher Project home page: https://github.com/daormar/debasher. Operating system(s): UNIX-like Programming language: Bash, Python Other requirements: Graphviz. License: GNU LGPL Any restrictions to use by non-academics: License needed. Additional code: The code used to generate the results of the experiments reported in the article is available at https://github.com/daormar/wfm-scalability-test

## Abbreviations

CBSE: Component-based software engineering
CWL: Common workflow language
DAG: Directed acyclic graph
FBP: Flow-based programming
FIFO: First-in First-out
HPC: High performance computing
IP: Information packets
OOP: Object oriented programming
SoC: Separation of concerns
TB: Terabyte
WDL: Workflow description language
WfM: Workflow manager

## Footnotes

1 https://slurm.schedmd.com

2 https://www.commonwl.org/specification/

3 https://openwdl.org/spec/

4 https://github.com/common-workflow-language/cwl-v1.3

5 https://github.com/ecmwf/ecflow

6 https://github.com/nextflow-io/nextflow/issues/1477
