# Supplementary material for: DeBasher: a flow-based programming bash extension for the implementation of complex and interactive workflows with stateful processes
Source: BMC Bioinformatics. 2025 Apr 16;26:106. doi: 10.1186/s12859-025-06108-1 (PMC12004750; doi:10.1186/s12859-025-06108-1)
Supplement: Supplementary file 1 — Supplementary Material 1. [file 12859_2025_6108_MOESM1_ESM.pdf]

DeBasher: a Flow-Based Programming Bash Extension for the Implementation of Complex and Interactive Workflows with Stateful Processes

Daniel Ortiz-Martínez[1]

[1]Department of Mathematics and Computer Science, Universitat de Barcelona, Gran Via de les Corts Catalanes, 585, 08007, Barcelona, Spain

Supplementary Materials

Contents

1 Sample Debasher Program **3**

2 Sample Debasher Program Using Python, R, Perl and Groovy **4**

3 The Telegram Problem **6**

4 Telegram Problem Implementation with DeBasher **6**
4.1 Original Telegram Problem 6
4.2 Telegram Problem With Two Processes 10
4.3 Telegram Problem With Two Job Steps 11
4.4 Array of Telegram Problems 13
4.5 Imperative Telegram Problem 14

5 Telegram Problem Implementation with Other Tools **15**
5.1 CWL 16
5.2 WDL 17
5.3 Nextflow 17
5.4 Bpipe 18
5.5 Snakemake 19
5.6 Toil 19

6 Programs with Arrays: a Code Reuse Example **20**

7 Network Reconfiguration Example **21**

8 Programs with Cycles **23**

9 Dynamic Scheduling Example **25**

10 User-Defined Triggers and Interactive Programs **26**

11 Runtime Piping **27**

12 Scalability Experiments **29**
12.1 Experiments Configuration 29
12.2 Results 30

13 Sample Bioinformatics Workflow **32**

References **35**

1 Sample Debasher Program

To illustrate how a DeBasher program looks like, Figure S1 shows the implementation of the "Hello World!" program[1]. Extended information about creating programs with DeBasher can be found in its technical documentation[2]. The code has four functions:

- **`hello_world_explain_cmdline_opts`**: this function is used to describe or document the options of the `hello_world` process, and more specifically, those that should be specified through the command line by the user. In this case, the process accepts only the `-s` option, which allows to specify the string to be displayed by the program.

- **`hello_world_define_opts`**: this function should establish the options passed to the `hello_world` function (this differs from the previous function, whose purpose was just to document command line options). In this case, the `-s` option provided in the command line by the user will be passed to the process. If no option is provided, then the "Hello World!" string is used as a default value.

- **`hello_world`**: the `hello_world` function implements the process of the same name. It reads the option defined by the `hello_world_define_opts` function and print it to the standard output.

- **`debasher_hello_world_program`**: this function defines the program implemented by the module. In particular, the program executes only the `hello_world` process.

```
hello_world_explain_cmdline_opts()
{
    # -s option
    local description="String to be displayed ('Hello World!' by default)"
    explain_cmdline_opt "-s" "<string>" "$description"
}

hello_world_define_opts()
{
    # Initialize variables
    local cmdline=$1
    local optlist=""

    # Obtain value of -s option
    local str=$(get_cmdline_opt "${cmdline}" "-s")

    # -s option
    if [ "${str}" = "${OPT_NOT_FOUND}" ]; then
        define_opt "-s" "Hello World!" optlist || return 1
    else
        define_opt "-s" "$str" optlist || return 1
    fi

    # Save option list
    save_opt_list optlist
}

hello_world()
{
    # Initialize variables
    local str=$(read_opt_value_from_func_args "-s" "$@")

    # Show message
    echo "${str}"
}

debasher_hello_world_program()
{
    add_debasher_process "hello_world" "cpus=1 mem=32 time=00:01:00"
}
```

Figure S1: DeBasher implementation of "Hello World!" program.

DeBasher incorporates functionality to graphically represent the network associated to a given program. Figure S2 shows the graph for the "Hello World!" program, which contains only one process (represented by a rectangle) with one input option (represented by an ellipse). A gray box is used to enclose the two elements.

[1] Note: all of the diagrams shown in this document were automatically generated with DeBasher.

[2] https://debasher.readthedocs.io/en/latest/

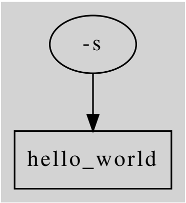

Figure S2: Process graph for the “Hello World!” DeBasher program.

2 Sample Debasher Program Using Python, R, Perl and Groovy

Due to the fact that FBP is language agnostic, process implementation can be done in multiple languages. For this purpose, it is necessary to store the code in a variable with a specific name, that results from concatenating the name of the process and the extension of the files used by the language. The code should ensure that the input and output options of the specific process are read and carefully processed.

Figure S3 shows the implementation of the `hello_world` process using Python, R, Perl and Groovy. To build the complete “Hello World!” program, it would only be necessary to replace the `hello_world` function in Figure S1 by the implementation in the desired language.

Python

```
hello_world_py=$(cat <<'EOF'
import argparse

# Create the parser
parser = argparse.ArgumentParser()

# Add the "-s" option with an integer argument
parser.add_argument('-s', type=str, required=True, help='String to be displayed')

# Parse the arguments
args = parser.parse_args()

# Access the value of "-s"
s = args.s

# Print message
print(s)
EOF
)
```

R

```
hello_world_r=$(cat <<'EOF'
args <- commandArgs(trailingOnly = TRUE)

# Function to parse arguments
parse_args <- function(args) {
  options <- list()
  i <- 1
  while (i <= length(args)) {
    if (args[i] == "-s") {
      if ((i + 1) <= length(args)) {
        options$string <- args[i + 1]
        i <- i + 1
      } else {
        stop("Option -s requires a string argument.")
      }
    }
    i <- i + 1
  }
  return(options)
}

# Parse the command-line arguments
options <- parse_args(args)

# Ensure the string was provided
if (is.null(options$string)) {
  stop("You must provide a string with the -s option.")
}

# Print the string
print(options$string)
EOF
)
```

Perl

```
hello_world_perl=$(cat <<'EOF'
use strict;
use warnings;
use Getopt::Long;

# Variables to hold command-line options
my $string;

# Parse command-line options
GetOptions("s=s" => \$string) or die "Error in command line arguments\n";

# Ensure the string was provided
die "You must provide a string with the -s option.\n" unless $string;

# Print string
print "$string\n";
EOF
)
```

Groovy

```
hello_world_groovy=$(cat <<'EOF'
def parseArgs(args) {
    def options = [:]
    args.eachWithIndex { arg, index ->
        if (arg == '-s' && index < args.size() - 1) {
            options.string = args[index + 1]
        }
    }
    if (!options.string) {
        throw new IllegalArgumentException("You must provide a string with the -s option.")
    }
    return options
}

def options = parseArgs(this.args)

println "${options.string}"
EOF
)
```

Figure S3: Implementation details of the `hello_world` process using Python, R, Perl and Groovy.

3 The Telegram Problem

The Telegram problem is used in [4] to illustrate how FBP programs are designed. More specifically, it involves creating a program that takes input lines of text and produces output lines with maximum word count, ensuring that each line's character count remains under a specified limit. Words must remain intact without splitting, and it is assumed that no word exceeds the line's length.

Figure S4 shows a Bash implementation of the Telegram problem using two functions, one to decompose the input text into words (`decompose`) and another one that takes the words and generates lines with a given maximum length (`recompose`). Both functions internally use the UNIX `awk` command for text processing. Once the two functions have been defined, they are combined by means of a Bash pipeline.

```
decompose()
{
    local inf=$1

    awk '{for(i=1;i<=NF;++i) print $i}' "${inf}"
}

recompose()
{
    local char_lim=$1

    awk -v maxlen="${char_lim}" 'BEGIN{len=0}
             {
              if(len + length($0) <= char_lim)
              {
                if(len > 0) printf" "
                printf"%s", $0
                len = len + length($0)
              }
              else
              {
                printf"\n%s",$0
                len = length($0)
              }
              if(len+1 <= char_lim)
               len = len + 1
             }'
}

inf=$1
char_lim=$2

decompose "${inf}" | recompose "${char_lim}"
```

Figure S4: Bash implementation of the Telegram problem.

4 Telegram Problem Implementation with DeBasher

This section describes five possible ways to implement the Telegram problem using DeBasher.

4.1 Original Telegram Problem

The original implementation of the Telegram problem from an FBP perspective was proposed in [4]. In particular, the author mentions the necessity of defining four processes that are depicted in Figure S5. Those processes are the following:

- `rseq`: this process takes a file as input (`-f` option) and reads it line by line, sending the file content to the `decomposer` process.
- `decomposer`: this process fragments the content of the input file and fragments it into words that are sent to the `recomposer` process.
- `recomposer`: the `recomposer` process takes as input the stream of words composing the input file provided by the `decomposer` process and the character limit per each line (`-c` option) and generates a stream of text lines whose length is below the character limit. This stream is sent to the `wseq` process.

- `wseq`: finally, the `wseq` process takes the stream of text generated by the `recomposer` process and writes it to a file.

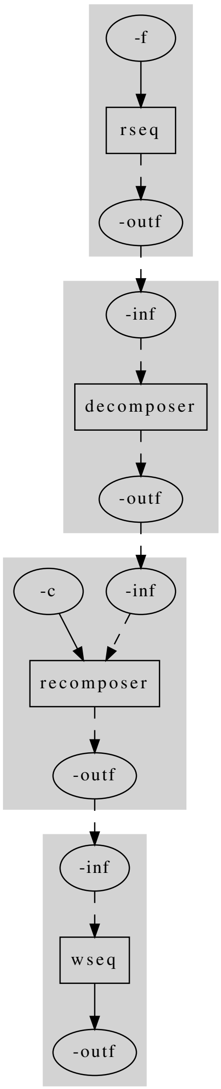

Figure S5: Process graph for the original formulation of the Telegram problem in the context of FBP.

Figure S6 shows the DeBasher code implementing the processes mentioned above for the original implementation of the Telegram problem from an FBP perspective. It is worthy of note that the `decomposer` and `recomposer` code that was shown in Figure S4 for the standard Bash implementation is used here without any modification.

On the other hand, Figure S7 shows how the options for each process are defined. The `define_opt_from_proc_out` function plays a fundamental role to connect a given process with another one. Dashed lines denote a connection between processes made by means of a pipeline.

Finally, once the different processes with their respective options have been defined, combining them into a program is straightforward, as it is shown in Figure S8.

```
rseq()
{
    # Initialize variables
    local outf=$(read_opt_value_from_func_args "-outf" "$@")
    local file=$(read_opt_value_from_func_args "-f" "$@")

    # Read sequence
    cat "${file}" > "${outf}" || return 1
}

decomposer()
{
    # Initialize variables
    local inf=$(read_opt_value_from_func_args "-inf" "$@")
    local outf=$(read_opt_value_from_func_args "-outf" "$@")

    # Decompose input
    awk '{for(i=1;i<=NF;++i) print $i}' "${inf}" > "${outf}" ; pipe_fail || return 1
}

recompose()
{
    local char_lim=$1
    local file=$2

    awk -v char_lim="${char_lim}" 'BEGIN{len=0}
             {
              if(len + length($0) <= char_lim)
              {
                if(len > 0) printf" "
                printf"%s", $0
                len = len + length($0)
              }
              else
              {
                printf"\n%s",$0
                len = length($0)
              }
              if(len+1 <= char_lim)
               len = len + 1
             }' "${file}"
}

recomposer()
{
    # Initialize variables
    local char_lim=$(read_opt_value_from_func_args "-c" "$@")
    local inf=$(read_opt_value_from_func_args "-inf" "$@")
    local outf=$(read_opt_value_from_func_args "-outf" "$@")

    # Recompose input
    recompose "${char_lim}" "${inf}" > "${outf}" ; pipe_fail || return 1
}

wseq()
{
    # Initialize variables
    local outf=$(read_opt_value_from_func_args "-outf" "$@")
    local inf=$(read_opt_value_from_func_args "-inf" "$@")

    # Write sequence
    cat "${inf}" > "${outf}" || return 1
}
```

Figure S6: Process implementation for the original formulation of the Telegram problem in the context of FBP.

```
rseq_define_opts()
{
    # Initialize variables
    local cmdline=$1
    local optlist=""

    # Define option for rseq FIFO
    local fifoname="rseq_fifo"
    define_fifo_opt "-outf" "${fifoname}" optlist || return 1

    # -f option
    define_cmdline_opt "$cmdline" "-f" optlist || return 1

    # Save option list
    save_opt_list optlist
}

decomposer_define_opts()
{
    # Initialize variables
    local cmdline=$1
    local optlist=""

    # -inf option
    define_opt_from_proc_out "-inf" "rseq" "-outf" optlist || return 1

    # Define option for decomposer FIFO
    fifoname="dc_fifo"
    define_fifo_opt "-outf" "${fifoname}" optlist || return 1

    # Save option list
    save_opt_list optlist
}

recomposer_define_opts()
{
    # Initialize variables
    local cmdline=$1
    local optlist=""

    # -c option
    define_cmdline_opt "$cmdline" "-c" optlist || return 1

    # -inf option
    define_opt_from_proc_out "-inf" "decomposer" "-outf" optlist || return 1

    # Define option for decomposer FIFO
    fifoname="rc_fifo"
    define_fifo_opt "-outf" "${fifoname}" optlist || return 1

    # Save option list
    save_opt_list optlist
}

wseq_define_opts()
{
    # Initialize variables
    local cmdline=$1
    local process_spec=$2
    local process_name=$3
    local process_outdir=$4
    local optlist=""

    # Define name of output file
    local outf="${process_outdir}/output.txt"
    define_opt "-outf" "${outf}" optlist || return 1

    # -inf option
    define_opt_from_proc_out "-inf" "recomposer" "-outf" optlist || return 1

    # Save option list
    save_opt_list optlist
}
```

Figure S7: Option definition for the original formulation of the Telegram problem in the context of FBP.

```
debasher_telegram_program()
{
    add_debasher_process "rseq"        "cpus=1 mem=32 time=00:05:00"
    add_debasher_process "decomposer"  "cpus=1 mem=32 time=00:05:00"
    add_debasher_process "recomposer"  "cpus=1 mem=32 time=00:05:00"
    add_debasher_process "wseq"        "cpus=1 mem=32 time=00:05:00"
}
```

Figure S8: Program definition for the original formulation of the Telegram problem in the context of FBP.

4.2 Telegram Problem With Two Processes

The Telegram problem can be redefined using only two processes, the `decomposer` and the `recomposer` processes. This alternative is less modular than the one shown in the previous section, since the `rseq` and the `wseq` processes are no longer independent. However, this implementation strategy makes more sense for worfklow managers that are not able to handle real pipelines (for instance, if regular files were used, `rseq` would just copy the input file into another one).

Figure S9 shows a process diagram, where the two processes that are defined are connected by means of a pipeline denoted by a dashed line.

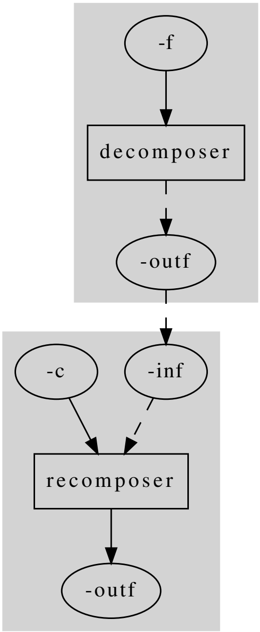

Figure S9: Process graph for the Telegram problem with two processes.

It is illustrative to show an alternative graph expressing the execution process dependencies. Given that both processes are connected by a pipeline, they will start execution simultaneously, which is reflected in the dependency graph shown in Figure S10.

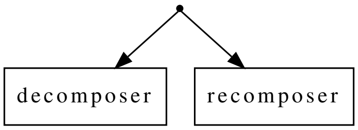

Figure S10: Dependency graph for the Telegram problem with two processes.

When implementing the Telegram problem with two processes using DeBasher, the previous process implementation given in Figure S6 needs no modification. Program definition is straightforward, since it is only necessary to add the `decomposer` and `recomposer` processes. What would need a more substantial modification would be the option definition code. This code can be seen in Figure S11.

```
decomposer_define_opts()
{
    # Initialize variables
    local cmdline=$1
    local optlist=""

    # Define option for decomposer FIFO
    local fifoname="dc_fifo"
    define_fifo_opt "-outf" "${fifoname}" optlist || return 1

    # -f option
    define_cmdline_opt "$cmdline" "-f" optlist || return 1

    # Save option list
    save_opt_list optlist
}

recomposer_define_opts()
{
    # Initialize variables
    local cmdline=$1
    local process_spec=$2
    local process_name=$3
    local process_outdir=$4
    local optlist=""

    # Define name of output file
    local outf="${process_outdir}/output.txt"
    define_opt "-outf" "${outf}" optlist || return 1

    # -c option
    define_cmdline_opt "$cmdline" "-c" optlist || return 1

    # Define option for decomposer FIFO
    define_opt_from_proc_out "-inf" "decomposer" "-outf" optlist || return 1

    # Save option list
    save_opt_list optlist
}
```

Figure S11: Option definition for the Telegram problem with two processes.

4.3 Telegram Problem With Two Job Steps

The Telegram problem implementation with two processes given in the previous section can be modified so as to work with files instead of pipelines, resulting in a very similar process graph given in Figure S12, where the dashed line connecting the `decomposer` and `recomposer` processes is replaced by a continuous one.

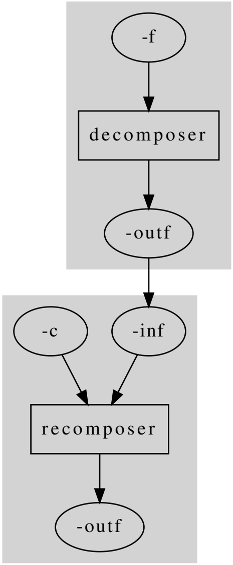

Figure S12: Process graph for the Telegram problem with two job steps.

When replacing the pipeline used to connect both processes by a regular file, the `recomposer` process can only start execution after the `decomposer` process has finished. This is graphically expressed in the dependency graph shown in Figure S13. The arc connecting the `decomposer` process to the `recomposer` process is labeled with `afterok` to specify that the latter will only start after the successful execution of the former.

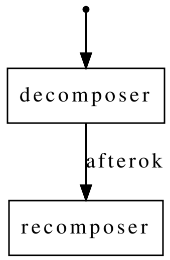

Figure S13: Dependency graph for the Telegram problem with two job steps.

The DeBasher implementation of the Telegram problem with two job steps can be made just by changing the code that defines the process options, as it is shown in Figure S14. This property of easily replacing processes by job steps is highlighted in [4] as one defining feature of FBP.

```
decomposer_define_opts()
{
    # Initialize variables
    local cmdline=$1
    local process_spec=$2
    local process_name=$3
    local process_outdir=$4
    local optlist=""

    # -f option
    define_cmdline_opt "$cmdline" "-f" optlist || return 1

    # Define name of output file
    local outf="${process_outdir}/words.txt"
    define_opt "-outf" "${outf}" optlist || return 1

    # Save option list
    save_opt_list optlist
}

recomposer_define_opts()
{
    # Initialize variables
    local cmdline=$1
    local process_spec=$2
    local process_name=$3
    local process_outdir=$4
    local optlist=""

    # -c option
    define_cmdline_opt "$cmdline" "-c" optlist || return 1

    # -inf option
    define_opt_from_proc_out "-inf" "decomposer" "-outf" optlist || return 1

    # Define name of output file
    local outf="${process_outdir}/output.txt"
    define_opt "-outf" "${outf}" optlist || return 1

    # Save option list
    save_opt_list optlist
}
```

Figure S14: Option definition for the Telegram problem with two job steps.

4.4 Array of Telegram Problems

The Telegram problem with two processes/job steps can be easily defined as an array, that is, we can execute an arbitrary number of `decomposer` and `recomposer` process pairs. Figure S15 shows the corresponding process graph, assuming that the array size is equal to 2.

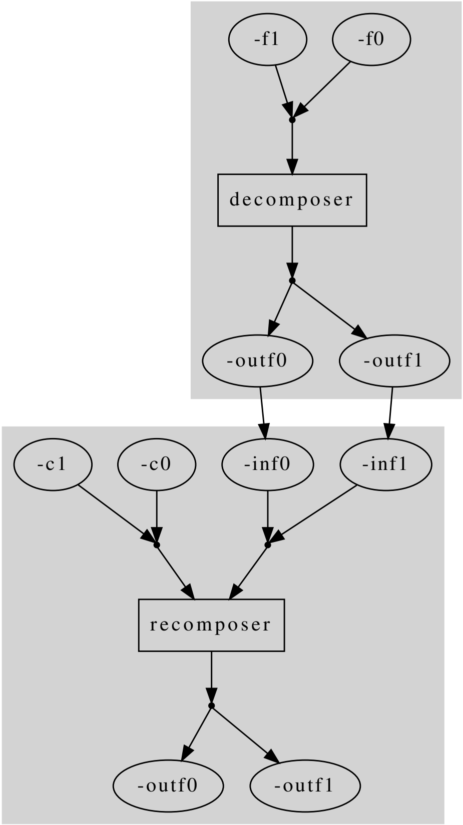

Figure S15: Process graph for an array of Telegram problems.

In order to implement an array of Telegram problems, it is only required to modify process option definition with respect to the initial implementation. Figure S16 shows how the `define_opts` method is redefined for the `decomposer` and `recomposer` processes. Specifically, the option lists for the different instances of the Telegram problem will be stored in an array. The code executes a `for` loop to populate these arrays.

```
decomposer_define_opts()
{
    # Initialize variables
    local cmdline=$1
    local process_spec=$2
    local process_name=$3
    local process_outdir=$4
    local optlist=""

    # Obtain value of -f option
    pref_of_files=$(get_cmdline_opt "${cmdline}" "-f")

    # Process files
    local i=0
    for file in "${pref_of_files}"*; do
        local specific_optlist=${optlist}

        # Define name of input file
        define_opt "-f" "${file}" specific_optlist || return 1

        # Define name of output file
        local outf="${process_outdir}/words_$i.txt"
        define_opt "-outf" "${outf}" specific_optlist || return 1

        # Save option list
        save_opt_list specific_optlist

        # Increase index
        i=$((i + 1))
    done
}

recomposer_define_opts()
{
    # Initialize variables
    local cmdline=$1
    local process_spec=$2
    local process_name=$3
    local process_outdir=$4
    local optlist=""

    # Obtain value of -f option
    pref_of_files=$(get_cmdline_opt "${cmdline}" "-f")

    # -c option
    define_cmdline_opt "$cmdline" "-c" optlist || return 1

    # Process files
    local i=0
    for file in "${pref_of_files}"*; do
        local specific_optlist=${optlist}

        # -inf option
        define_opt_from_proc_task_out "-inf" "decomposer" "$i" "-outf" specific_optlist || return 1

        # Define name of output file
        local outf="${process_outdir}/output_$i.txt"
        define_opt "-outf" "${outf}" specific_optlist || return 1

        # Save option list
        save_opt_list specific_optlist

        # Increase index
        i=$((i + 1))
    done
}
```

Figure S16: Option definition for an array of Telegram problems.

4.5 Imperative Telegram Problem

The Telegram problem can be implemented by means of DeBasher using only one process that internally calls the previously defined `decomposer` and `recomposer` processes in a purely imperative way. The corresponding process graph is shown in Figure S17, where only one process called `telegram` is defined.

The ability to execute processes in an imperative way from another one is exclusive to DeBasher and is not incorporated into other tools mentioned in this study.

The DeBasher implementation of the imperative Telegram problem requires defining the `telegram` process and its options, as it is shown in Figure S18. The `telegram` process calls the `decomposer` and `recomposer` processes imperatively by means of the `seq_execute` function (the name stands for *sequential execute*). The code of the called processes does not need to be redefined.

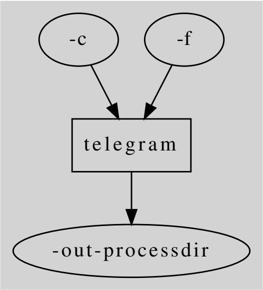

Figure S17: Process graph for the imperative Telegram problem.

```
telegram_define_opts()
{
    # Initialize variables
    local cmdline=$1
    local process_spec=$2
    local process_name=$3
    local process_outdir=$4
    local optlist=""

    # Define the -out-processdir option, the output directory for the process
    define_opt "-out-processdir" "${process_outdir}" optlist || return 1

    # -f option
    define_cmdline_opt "$cmdline" "-f" optlist || return 1

    # -c option
    define_cmdline_opt "$cmdline" "-c" optlist || return 1

    # Save option list
    save_opt_list optlist
}

telegram()
{
    # Initialize variables
    local outd=$(read_opt_value_from_func_args "-out-processdir" "$@")
    local file=$(read_opt_value_from_func_args "-f" "$@")
    local char_lim=$(read_opt_value_from_func_args "-c" "$@")

    # Execute decomposer
    seq_execute decomposer -f "${file}" -outf "${outd}"/words.txt || return 1

    # Obtain number of lines of decomposer output
    local nlines=$(wc -l "${outd}"/words.txt | awk '{print $1}')

    if [ "${nlines}" -eq 0 ]; then
        echo "Warning: Decomposer's output is empty" >&2
        echo -n "${outd}"/output.txt || return 1
    else
        # Execute recomposer
        seq_execute recomposer -c "${char_lim}" -inf "${outd}"/words.txt -outf "${outd}"/output.txt ||
            return 1
    fi
}
```

Figure S18: Option definition and process implementation for the imperative Telegram problem.

5 Telegram Problem Implementation with Other Tools

It is interesting to inspect how the Telegram problem can be implemented with other tools. All of the tools considered in this section are tools specialized in the execution of workflows. From all the Telegram problem versions described above, we will focus on the one using job steps described in Section 4.3, since the all of the tools used allow to implement it.

5.1 CWL

The Common Workflow Language[3] (CWL), is not a particular tool but a language specification for which different implementations have been defined.

CWL distributes the code of the `decomposer` and `recomposer` processes in different files, as it is shown in Figure S19. CWL is very different with respect to other workflow engines because it only allows to execute one shell-script command per workflow step.

`decomposer` File

```
baseCommand: [awk, '{for(i=1;i<=NF;++i) print $i}']
stdout: words.txt

inputs:
  input_file:
    type: File
    inputBinding:
      position: 1

outputs:
  decomposer_out:
    type: stdout
```

`recomposer` File

```
baseCommand: [awk,-v]
stdout: output.txt

inputs:
  char_lim:
    type: int
    inputBinding:
      prefix: char_lim=
      separate: false
      position: 1

  pattern:
    type: string
    default: 'BEGIN{len=0}
             {
              if(len + length($0) <= char_lim)
              {
                if(len > 0) printf" ";
                printf"%s", $0;
                len = len + length($0);
              }
              else
              {
                printf"\n%s",$0;
                len = length($0);
              }
              if(len+1 <= char_lim)
               len = len + 1;
             }'
    inputBinding:
      position: 2

  words_file:
    type: File
    inputBinding:
      position: 3

outputs:
  recomposer_out:
    type: stdout
```

`Workflow` File

```
inputs:
  input_file: File
  char_lim: int

steps:
  decomposer:
    run: telegram.decomposer.tool.cwl
    in:
      input_file: input_file
    out: [decomposer_out]
  recomposer:
    run: telegram.recomposer.tool.cwl
    in:
      words_file: decomposer/decomposer_out
      char_lim: char_lim
    out: [recomposer_out]

outputs:
  output:
    type: File
    outputSource: recomposer/recomposer_out
```

Figure S19: Implementation details of the Telegram problem with two processes using CWL.

[3] https://www.commonwl.org/specification/

5.2 WDL

The Workflow Description Language[4] (WDL) is similar to CWL in the sense that is not a particular tool but a language specification.

WDL uses `command` blocks to incorporate shell-scripting code, as it can be seen in Figure S20.

```
task Decomposer {
  File input_file

  command <<<
    awk '{for(i=1;i<=NF;++i) print $i}' ${input_file} > words.txt
  >>>

  output {
    File words_file = "words.txt"
  }
}

task Recomposer {
  File input_file
  Int char_lim

  command <<<
    awk -v maxlen=${char_lim} 'BEGIN{len=0}
             {
              if(len + length($0) <= char_lim)
              {
                if(len > 0) printf" ";
                printf"%s", $0;
                len = len + length($0);
              }
              else
              {
                printf"\n%s",$0;
                len = length($0);
              }
              if(len+1 <= char_lim)
               len = len + 1;
             }' ${input_file} > output.txt
  >>>

  output {
    File output_file = "output.txt"
  }
}

workflow Telegram {
  File input_file
  Int char_lim

  call Decomposer {
   input:
    input_file= input_file
  }

  call Recomposer {
   input:
    input_file= Decomposer.words_file,
    char_lim= char_lim
  }

  output {
    File output_file = Recomposer.output_file
  }
}
```

Figure S20: Implementation details of the Telegram problem with two processes using WDL.

5.3 Nextflow

Nextflow [2] is a very successful and well known workflow manager using Groovy as a coordination language.

Figure S21 shows the implementation of both processes involved in the Telegram problem. Again there are specific blocks devoted to the execution of shell-script code.

[4] https://openwdl.org/spec/

```
process decomposer {
  input:
    path file

  output:
    path "decompose.out"

    """
    cat < ${file} | awk '{for(i=1;i<=NF;++i) print \$i}' > decompose.out
    """
}

process recomposer {
  input:
    path decompose_out

  output:
    path "recompose.out"

    """
    awk -v char_lim=$params.char_lim 'BEGIN{len=0}
             {
              if(len + length(\$0) <= char_lim)
              {
                if(len > 0) printf" "
                printf"%s", \$0
                len = len + length(\$0)
              }
              else
              {
                printf"\\n%s",\$0
                len = length(\$0)
              }
              if(len+1 <= char_lim)
               len = len + 1
             }' ${decompose_out} > recompose.out
    """
}

workflow {
   def ch = Channel.fromPath(params.file)
   decompose(ch) | recompose
}
```

Figure S21: Implementation details of the Telegram problem with two processes using Nextflow.

5.4 Bpipe

Bpipe [5] is a platform to execute workflows which uses Groovy as the coordination language. In this regard, it is similar to Nextflow, and indeed, Nextflow was born as a branch of Bpipe according to the Bpipe creators.

Figure S22 shows the Bpipe code implementing both the `decomposer` and `recomposer` processes (or more accurately, job steps). In the code, `exec` blocks allows to execute shell-script code.

```
decomposer = {
    exec """
    cat < $input | awk '{for(i=1;i<=NF;++i) print \$i}' > $output
    """
}

recomposer = {
    exec """
    awk -v char_lim=${CHAR_LIM} 'BEGIN{len=0}
             {
              if(len + length(\$0) <= char_lim)
              {
                if(len > 0) printf" ";
                printf"%s", \$0;
                len = len + length(\$0);
              }
              else
              {
                printf"\\n%s",\$0;
                len = length(\$0);
              }
              if(len+1 <= char_lim)
               len = len + 1;
             }' $input > $output
    """
}

run {
    decompose + recompose
}
```

Figure S22: Implementation details of the Telegram problem with two processes using Bpipe.

5.5 Snakemake

Snakemake [3] is a well established workflow execution tool. Snakemake follows a very different approach when compared to other tools such as Nextflow or Bpipe. In particular, it is inspired by the well known UNIX `make` tool. The `make` tool is typically used when compiling large C programs, resolving file dependencies and issuing commands to carry out the required recompilations. Snakemake follows a similar strategy to implement workflows composed of multiple processes.

Figure S23 shows the definition of the `decomposer` and `recomposer` processes using the Snakemake syntax. Again, the tool has a specific block to provide shell-script code.

```
rule decomposer:
     input:
        "data/data_1M.txt"
     output:
        "results/words.txt"
     shell:
        "cat {input} | awk '{{for(i=1;i<=NF;++i) \
                                {{print $i}} }}' > {output}"

rule recomposer:
     input:
        "results/words.txt"
     output:
        "results/output.txt"
     shell:
        "awk -v char_lim=20 '{{ \
          current_line = current_line $0 \
          if (length(current_line) < char_lim) \
           current_line = current_line " " \
          else {{ \
           print current_line \
           current_line = $0 \
          }} \
         }} \
        END {{ \
          if (length(current_line) > 0) \
           print current_line \
        }}' {input} > {output}"
```

Figure S23: Implementation details of the Telegram problem with two processes using Snakemake.

5.6 Toil

Toil [7] is a workflow engine that can operate in different ways, supporting CWL and WDL (see more details in Sections 5.1 and 5.2, respectively). However, Toil's native operation mode uses pure Python. This is reflected in Figure S24, where the code of the `decomposer` and `recomposer` processes is shown.

```python
def decomposer(job, infid):
    with job.fileStore.readGlobalFileStream(infid, encoding='utf-8') as fi:
        with job.fileStore.writeGlobalFileStream(encoding='utf-8') as (fo, decfid):
            for line in fi:
                line = line.strip()
                words = line.split()
                for word in words:
                    print(word, file=fo)
    return decfid

def recomposer(job, decfid, char_lim=20):
    with job.fileStore.readGlobalFileStream(decfid, encoding='utf-8') as fi:
        with job.fileStore.writeGlobalFileStream(encoding='utf-8') as (fo, recfid):
            length = 0
            for line in fi:
                word = line.strip()
                if(length + len(word) <= char_lim):
                    if length > 0:
                        print(" ", sep='', end='', file=fo)
                    print(word, sep='', end='', file=fo)
                    length = length + len(word)
                else:
                    print("\n", word, sep='', end='', file=fo)
                    length = len(word)
                if(length+1 <= char_lim):
                    length = length + 1
    return recfid

def telegram(job, infid, char_lim):
    decfid = decompose(job, infid)
    j = job.addChildJobFn(recompose, decfid, char_lim)
    return j.rv()
```

Figure S24: Implementation details of the Telegram problem with two processes using Toil.

6 Programs with Arrays: a Code Reuse Example

DeBasher easily enables the definition of arrays of processes as we saw for the array of Telegram problems example in Section 4.4. In this section we show an alternative way to define a program incorporating an array of processes.

Let us consider a very simple program composed of only one process called `host1` that receives an identifier as input and executes the `hostname` UNIX command, printing the result to the standard output. We will refer to this program as `debasher_host_process`. Figure S25 shows the required DeBasher code.

```
host1_define_opts()
{
    # Initialize variables
    local optlist=""

    # -id option
    define_opt "-id" 0 optlist || return 1

    # Save option list
    save_opt_list optlist
}

host1()
{
    # Initialize variables
    local id=$(read_opt_value_from_func_args "-id" "$@")

    # Show host name
    local hname=$(hostname)
    echo "${id}: ${hname}"
}

debasher_host_process_program()
{
    add_debasher_process "host1" "cpus=1 mem=32 time=00:10:00 throttle=64"
}
```

Figure S25: Code of the `debasher_host_process` program.

In order to work with an array of `host1`, it is possible to modify the `define_opts` method defined above, so as to incorporate a `while` or `for` loop adding groups of options to the `optlist` variable (see Figure S16 for an example). Alternatively, we can also define the `generate_opts` method for the process, which returns the options corresponding to a given array index. When using `generate_opts`, it is also necessary to implement a method returning the array size called `generate_opts_size`.

Figure S26 shows the implementation of the `debasher_host_process` program, which executes an array of `host1` processes using the `generate_opts` method. Such method now defines the identifier to be passed to `host1` as the task index. The program uses the `load_debasher_module` function so as to load the `debasher_host_process` program that was described above. The program also executes the `add_debasher_program` function to execute `debasher_host_process` as a subprogram.

```
load_debasher_module "debasher_host_process"

host1_explain_cmdline_opts()
{
    # -n option
    local description="Number of array tasks"
    explain_cmdline_req_opt "-n" "<int>" "$description"
}

host1_generate_opts_size()
{
    # Initialize variables
    local cmdline=$1

    # -n option
    local n_opt=$(get_cmdline_opt "$cmdline" "-n")

    echo ${n_opt}
}

host1_generate_opts()
{
    # Initialize variables
    local cmdline=$1
    local process_spec=$2
    local process_name=$3
    local process_outdir=$4
    local task_idx=$5
    local optlist=""

    # -id option
    define_opt "-id" ${task_idx} optlist || return 1

    # Save option list
    save_opt_list optlist
}

debasher_host_process_program()
{
    add_debasher_program "debasher_host_process"
}
```

Figure S26: Code of the `debasher_host_process` program that executes a process array. Replacing a single process by an array does not require any modification in the `program` method.

7 Network Reconfiguration Example

This section shows how to reconfigure the connection pattern of a particular program. Let us define the `debasher_net_reconf_1` program composed of two processes: `process_a` and `process_b`. `process_a` takes a string as input and writes it to an output file. On the other hand, `process_b` takes the name of a file as input and prints its content to the standard input.

Figure S27 shows a graph for the `debasher_net_reconf_1` program, where the two processes are disconnected. The code related to the program is shown in Figure S28.

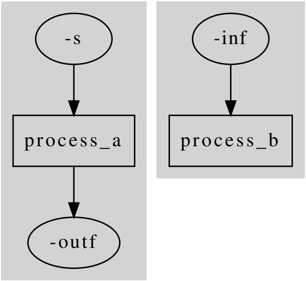

Figure S27: Process graph for the `debasher_net_reconf_1` program.

Now, let us suppose that we want to reconfigure the connection pattern of the `debasher_net_reconf_1` program. In particular, we need to connect the output of process `process_a` to the input of process `process_b`. We will refer to the new program as `debasher_net_reconf_2`, whose process graph is shown in Figure S29.

```
process_a_explain_cmdline_opts()
{
    # -s option
    local description="Input string"
    explain_cmdline_req_opt "-s" "<string>" "$description"
}

process_a_define_opts()
{
    # Initialize variables
    local cmdline=$1
    local process_spec=$2
    local process_name=$3
    local process_outdir=$4
    local optlist=""

    # -s option
    define_cmdline_opt "$cmdline" "-s" optlist || return 1

    # -outf option
    define_opt "-outf" "${process_outdir}/outf" optlist || return 1

    # Save option list
    save_opt_list optlist
}

process_a()
{
    # Initialize variables
    local str=$(read_opt_value_from_func_args "-s" "$@")
    local outf=$(read_opt_value_from_func_args "-outf" "$@")

    # Print string to file
    echo "${str}" > "${outf}"
}

process_b_explain_cmdline_opts()
{
    # -inf option
    local description="Input file"
    explain_cmdline_req_opt "-inf" "<str>" "$description"
}

process_b_define_opts()
{
    # Initialize variables
    local cmdline=$1
    local optlist=""

    # -inf option
    define_cmdline_opt "$cmdline" "-inf" optlist || return 1

    # Save option list
    save_opt_list optlist
}

process_b()
{
    # Initialize variables
    local inf=$(read_opt_value_from_func_args "-inf" "$@")

    # Print content of input file to the standard output
    cat "${inf}"
}

debasher_net_reconf_1_program()
{
    add_debasher_process "process_a" "cpus=1 mem=32 time=00:10:00"
    add_debasher_process "process_b" "cpus=1 mem=32 time=00:10:00"
}
```

Figure S28: DeBasher code for the `debasher_net_reconf_1` program.

Implementing the `debasher_net_reconf_2` program only requires to define a new module importing `debasher_net_reconf_1` and redefining the `define_opts` method for `process_b`. The resulting code is shown in Figure S30.

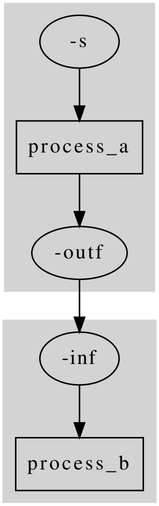

Figure S29: Process graph for the `debasher_net_reconf_2` program.

```
load_debasher_module "debasher_net_reconf_1"

process_b_explain_cmdline_opts()
{
    :
}

process_b_define_opts()
{
    # Initialize variables
    local optlist=""

    # -inf option
    define_opt_from_proc_out "-inf" "process_a" "-outf" optlist || return 1

    # Save option list
    save_opt_list optlist
}

debasher_net_reconf_2_program()
{
    add_debasher_program "debasher_net_reconf_1"
}
```

Figure S30: DeBasher code for the `debasher_net_reconf_2` program.

8 Programs with Cycles

DeBasher is the only tool among all those mentioned in this study that allows to implement programs with arbitrary cycles. This is due to the fact that FBP processes are in control of their life cycle, as opposed to the job steps implemented by other tools, which have a sequential nature and are executed reactively depending on the availability of the data they consume.

Figure S31 shows the graph of a program that contains a cycle. The program contains two processes, `master` and `worker`, where both are connected with pipelines.

Figure S32 shows the code of the processes. In particular, `master` uses a pipeline to send a numeric value to `worker`, and `worker` transforms the value, accumulates the result in an internal variable, and uses another pipeline to return the transformed value to `master`. More specifically, `worker` will add one to the value if the current cummulative value is below or equal to a threshold given by the user, or two otherwise. The cycle is repeated until `master` receives a value greater than another one given as input parameter. At the beginning of execution, `master` also receives the initial number for doing the calculations. Before terminating, `worker` prints the cummulative value to the standard output.

This simple example illustrates the ability of DeBasher to execute a program with cycles where one process maintains internal state information.

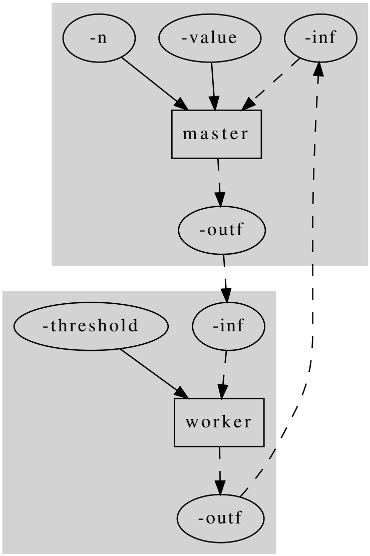

Figure S31: Process graph for a program with cycles.

```
master()
{
    # Initialize variables
    local n=$(read_opt_value_from_func_args "-n" "$@")
    local value=$(read_opt_value_from_func_args "-value" "$@")
    local inf=$(read_opt_value_from_func_args "-inf" "$@")
    local outf=$(read_opt_value_from_func_args "-outf" "$@")

    # Send value for transformation until is greater than n
    while [ "${value}" -le "${n}" ]; do
        echo "${value}" > "${outf}"
        echo "Sent value ${value}"
        value=$(cat "${inf}")
        echo "Received value ${value}"
        echo ""
    done

    # Send shutdown token
    echo "${SHUTDOWN_TOKEN}" > "${outf}"
}

worker()
{
    local threshold=$(read_opt_value_from_func_args "-threshold" "$@")
    local inf=$(read_opt_value_from_func_args "-inf" "$@")
    local outf=$(read_opt_value_from_func_args "-outf" "$@")

    # Execute loop until the shutdown token is received
    local sum=0
    while true; do
        value=$(cat "${inf}")
        echo "Received value ${value}"
        if [ "${value}" = "${SHUTDOWN_TOKEN}" ]; then
            break
        fi
        # Decide value increment depending on threshold
        if [ "${sum}" -le "${threshold}" ]; then
            value=$((value + 1))
        else
            value=$((value + 2))
        fi
        # Update sum
        sum=$((sum + value))

        echo "Transformed value ${value}"
        echo "Sum ${sum}"
        echo ""

        # Send current value
        echo "${value}" > "${outf}"
    done

    echo "Final value of sum: ${sum}"
}
```

Figure S32: Process code of the `debasher_cycle_state` program.

9 Dynamic Scheduling Example

In the example of the previous section, we saw how to implement a program with cycles, where one process communicated with another to perform transformations of a number given as input. However, the calculations were done locally. DeBasher allows to schedule dynamic processes in HPC infrastructure so as to carry out computations.

To illustrate this ability, we use exactly the same program structure, but the functions that make the number transformations are executed in Slurm. The new program is called `debasher_cycle_dyn_sched`. Figure S33 shows the required code for the two processes. One of the functions used to make the calculations is now implemented in Python.

```
master()
{
    # Initialize variables
    local n=$(read_opt_value_from_func_args "-n" "$@")
    local value=$(read_opt_value_from_func_args "-value" "$@")
    local inf=$(read_opt_value_from_func_args "-inf" "$@")
    local outf=$(read_opt_value_from_func_args "-outf" "$@")

    # Send value for transformation until is greater than n
    while [ "${value}" -le "${n}" ]; do
        echo "${value}" > "${outf}"
        echo "Sent value ${value}"
        value=$(cat "${inf}")
        echo "Received value ${value}"
        echo ""
    done

    # Send shutdown token
    echo "${SHUTDOWN_TOKEN}" > "${outf}"
}

transformation_a()
{
    local value=$1
    local outf=$2

    value=$((value + 1))
    echo ${value} > ${outf}
}

transformation_b_py=$(cat <<EOF
import sys
value = int(sys.argv[1])
fname = sys.argv[2]
with open(fname, 'w') as f:
    f.write(str(value + 2))
EOF
)

worker()
{
    local threshold=$(read_opt_value_from_func_args "-threshold" "$@")
    local inf=$(read_opt_value_from_func_args "-inf" "$@")
    local outf=$(read_opt_value_from_func_args "-outf" "$@")
    local outd=$(read_opt_value_from_func_args "-outd" "$@")

    # Execute loop until the shutdown token is received
    local sum=0
    while true; do
        # Read value
        local value=$(cat "${inf}")
        echo "Received value ${value}"
        if [ "${value}" = "${SHUTDOWN_TOKEN}" ]; then
            break
        fi

        # Decide transformation function depending on threshold
        if [ "${sum}" -le "${threshold}" ]; then
            seq_execute_slurm transformation_a "${value}" "${outd}/transformation_result.txt"
            value=$(cat "${outd}/transformation_result.txt")
        else
            seq_execute_slurm transformation_b_py "${value}" "${outd}/transformation_result.txt"
            value=$(cat "${outd}/transformation_result.txt")
        fi

        # Update sum
        sum=$((sum + value))

        echo "Transformed value ${value}"
        echo "Sum ${sum}"
        echo ""

        # Send current value
        echo "${value}" > "${outf}"
    done

    echo "Final value of sum: ${sum}"
}
```

Figure S33: Code for the processes of the `debasher_cycle_dyn_sched` program.

10 User-Defined Triggers and Interactive Programs

The program in the previous section can be modified so as to receive the initial value for `master` and the threshold for `worker` by means of FIFOs. This opens the possibility of implementing user-defined triggers and program interactivity. We will refer to this program as the `debasher_cycle_trigger_interactive` program. Figure S35 shows a process graph for the program, and the new code for the processes is shown in Figure S34.

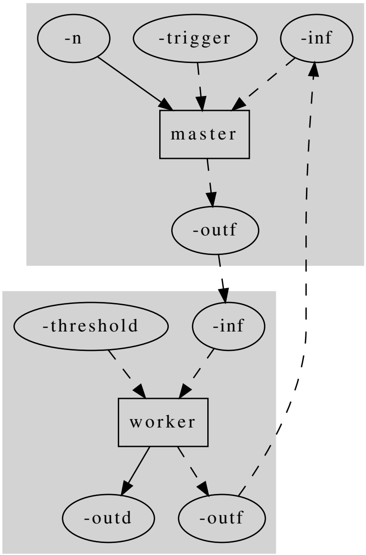

Figure S34: Process graph of a program with cycles with user-defined triggers and interactivity.

```
master()
{
    # Initialize variables
    local n=$(read_opt_value_from_func_args "-n" "$@")
    local trigger_fifo=$(read_opt_value_from_func_args "-trigger" "$@")
    local inf=$(read_opt_value_from_func_args "-inf" "$@")
    local outf=$(read_opt_value_from_func_args "-outf" "$@")

    # Set initial value
    local initial_value=$(cat "${trigger_fifo}")

    while [ "${initial_value}" != "${SHUTDOWN_TOKEN}" ]; do
        # Send value for transformation until is equal or greater than n
        local value="${initial_value}"
        echo "* Initial value ${value}"
        while [ "${value}" -le "${n}" ]; do
            echo "${value}" > "${outf}"
            echo "Sent value ${value}"
            value=$(cat "${inf}")
            echo "Received value ${value}"
            echo ""
        done

        # Set initial value
        local initial_value=$(cat "${trigger_fifo}")
    done

    # Send shutdown token
    echo "${SHUTDOWN_TOKEN}" > "${outf}"
}

transformation_a()
{
    local value=$1
    local outf=$2

    value=$((value + 1))
    echo ${value} > ${outf}
}

transformation_b()
{
    local value=$1
    local outf=$2

    value=$((value + 2))
    echo ${value} > ${outf}
}

worker()
{
    local threshold_fifo=$(read_opt_value_from_func_args "-threshold" "$@")
    local inf=$(read_opt_value_from_func_args "-inf" "$@")
    local outf=$(read_opt_value_from_func_args "-outf" "$@")
    local outd=$(read_opt_value_from_func_args "-outd" "$@")

    # Execute loop until the shutdown token is received
    local sum=0
    while true; do
        # Read value
        value=$(cat "${inf}")
        echo "Received value ${value}"
        if [ "${value}" = "${SHUTDOWN_TOKEN}" ]; then
            break
        fi

        # Read threshold
        local threshold=$(read_fifo_line "${threshold_fifo}")
        echo "Threshold value ${threshold}"

        # Decide transformation function depending on threshold
        if [ "${sum}" -le "${threshold}" ]; then
            seq_execute_slurm transformation_a "${value}" "${outd}/transformation_result.txt"
            value=$(cat "${outd}/transformation_result.txt")
        else
            seq_execute_slurm transformation_b "${value}" "${outd}/transformation_result.txt"
            value=$(cat "${outd}/transformation_result.txt")
        fi

        # Update sum
        sum=$((sum + value))

        echo "Transformed value ${value}"
        echo "Sum ${sum}"
        echo ""

        # Send current value
        echo "${value}" > "${outf}"
    done

    echo "Final value of sum: ${sum}"
}
```

Figure S35: Code for the processes of the `debasher_cycle_trigger_interactive` program.

11 Runtime Piping

Another interesting possibility is to connect two separate programs by using their FIFOs. The canonical example would be one program using a FIFO to produce its output, and another one using a FIFO to receive its input. Here we provide an example of such programs. For this purpose, we define the `debasher_counter`

program, which prints a count from 1 until another number given as input to an output FIFO. A process graph is given in Figure S36, and the process code can be seen in S37.

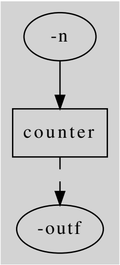

Figure S36: Process graph of a counter program.

```
counter_define_opts()
{
    # Initialize variables
    local cmdline=$1
    local optlist=""

    # -n option
    define_cmdline_opt "$cmdline" "-n" optlist || return 1

    # Define option for output FIFO
    local fifoname="counter_out"
    define_fifo_opt "-outf" "${fifoname}" optlist || return 1

    # Save option list
    save_opt_list optlist
}

counter()
{
    # Initialize variables
    local n=$(read_opt_value_from_func_args "-n" "$@")
    local outf=$(read_opt_value_from_func_args "-outf" "$@")

    # Increase value iteratively until it is greater than n
    local value=1
    while [ "${value}" -le "${n}" ]; do
        echo "${value}" > "${outf}"
        value=$((value + 1))
    done

    # Send shutdown token
    echo "${SHUTDOWN_TOKEN}" > "${outf}"
}
```

Figure S37: Code related to the `debasher_counter` program.

On the other hand, we define the `debasher_echo` program, which receives a FIFO stream as input and prints it to the standard output. A process graph is shown in Figure S38 and the corresponding code is given in Figure S39.

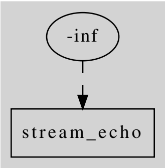

Figure S38: Process graph of an echo program.

Due to the fact that DeBasher exposes to the user all the FIFOs intervening in a program, it is possible to connect the two programs, for instance by using Bash commands. According to the process definitions, the names of the FIFOs to be connected would be `counter_out` for the `counter` process, and `stream_echo_in` for the `stream_echo` process.

```
stream_echo_define_opts()
{
    # Initialize variables
    local optlist=""

    # Define option for input FIFO
    local fifoname="stream_echo_in"
    define_fifo_opt "-inf" "${fifoname}" optlist || return 1

    # Save option list
    save_opt_list optlist
}

stream_echo()
{
    # Initialize variables
    local inf=$(read_opt_value_from_func_args "-inf" "$@")

    # Read input line by line
    local line
    while IFS= read -r line < "${inf}"; do
        if [ "${line}" = "${SHUTDOWN_TOKEN}" ]; then
            break
        else
            echo "${line}"
        fi
    done
}
```

Figure S39: Code related to the `debasher_echo` program.

12 Scalability Experiments

This section provides additional results to compare the ability to scale of the different tools tested in the study.

12.1 Experiments Configuration

The scalability experiments executed in this article are inspired in those reported in [1]. In that paper, a large set of instances of a one-step program (called `host_process`) or a two-step program (called `host_workflow`) is executed in an AWS Slurm ParallelCluster using different workflow managers.

The `host_process` program consists of a single process named `host1`, which internally runs the `hostname` UNIX command before terminating. Conversely, the `host_workflow` program executes both the `host1` and `host2` processes. Initially, it runs the `host1` process, identical to the one in the `host_process` program. Subsequently, it executes the `host2` process, which also runs the `hostname` command. For the rest of the experiments discussed in this section, we refer to a single execution of process `host1` or `host2` as a *workflow task*.

A DeBasher implementation of the `host_process` program has already been given in Section 6. However, our scalability experimentation is more focused on the two-step program. Figure S40 shows a process dependency diagram for the program, that we will call `debasher_host_workflow`. As it can be seen, the execution of `host2` depends on the previous execution of `host1`. During program execution, each workflow task (an instance of `host1` or `host2`) is assigned a numeric identifier, `id`. The arc connecting the two processes in Figure S40 is labeled with `aftercorr` to specify that the execution of the `host2` task with index `id` can start immediately after the `host1` task with the same index has successfully finished, that is, it has the same meaning as using the `aftercorr` dependency type in Slurm. The DeBasher implementation of both processes is shown in Figure S41. As it can be seen, both the `host1` and `host2` processes take an index as input and then execute the `hostname` UNIX command.

For our experiments, we implemented versions of the one- and two-step programs described above using CWL, WDL, Nextflow and DeBasher.

All the experiments were carried out in an AWS Slurm ParallelCluster with 1 head node and 32 computing nodes. Each node had 2 CPUs and 4GB of RAM. We used Toil [6] (v7.0.0) as execution engine for CWL, and Cromwell[5] (v87) for WDL. Nextflow (v24.04) comes with its own engine.

[5] https://github.com/broadinstitute/cromwell

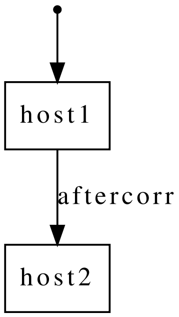

Figure S40: Process dependency graph for the `debasher_host_workflow` program.

```
host1()
{
    # Initialize variables
    local id=$(read_opt_value_from_func_args "-id" "$@")

    # Show host name
    local hname=$(hostname)
    echo "${id}: ${hname}"
}

host2()
{
    # Initialize variables
    local id=$(read_opt_value_from_func_args "-id" "$@")

    # Show host name
    local hname=$(hostname)
    echo "${id}: ${hname}"
}
```

Figure S41: Code for the `host1` and `host2` processes used in the `debasher_host_workflow` program.

12.2 Results

12.2.1 Overhead when Switching from Process to Workflow

One of the experiments carried out in [1], consists in measuring the time cost of executing the same number of workflow tasks with both the one- and the two-step programs. This allows to determine the overhead introduced by the execution engine to handle process dependencies for the two-step program.

Figure S42 shows the time cost of Nextflow and DeBasher when executing an increasing number of tasks for the two programs being considered. As it can be seen, there is an almost perfect overlap between the time costs of both programs for DeBasher, meaning that it does not introduce any meaningful overhead due to process dependency handling. In contrast, Nextflow does introduce some overhead when transitioning from `host_process` to `host_workflow`.

12.2.2 Task Distribution Across Nodes

The output of the `hostname` command for each task can be analyzed so as to get an idea of how well the tasks are distributed across the available cluster nodes. For this purpose, the output of all tasks is collected and the fraction of tasks executed at each node is computed. This fraction can be compared with the ideal one, where all tasks are equally distributed across nodes. Once the observed and ideal fractions of executed tasks have been calculated, we compute the mean absolute error (MAE) so as to get a quantitative measure of the deviation. A value of zero would mean that the ideal distribution was achieved.

Table S1 shows the MAE when executing `host_workflow` with 10K tasks by means of DeBasher and Nextflow. Nextflow was executed for increasing array sizes. As it can be seen in the table, DeBasher obtained the lowest MAE, indicating a better distribution of tasks across nodes with respect to Nextflow, no matter the size of the array chosen.

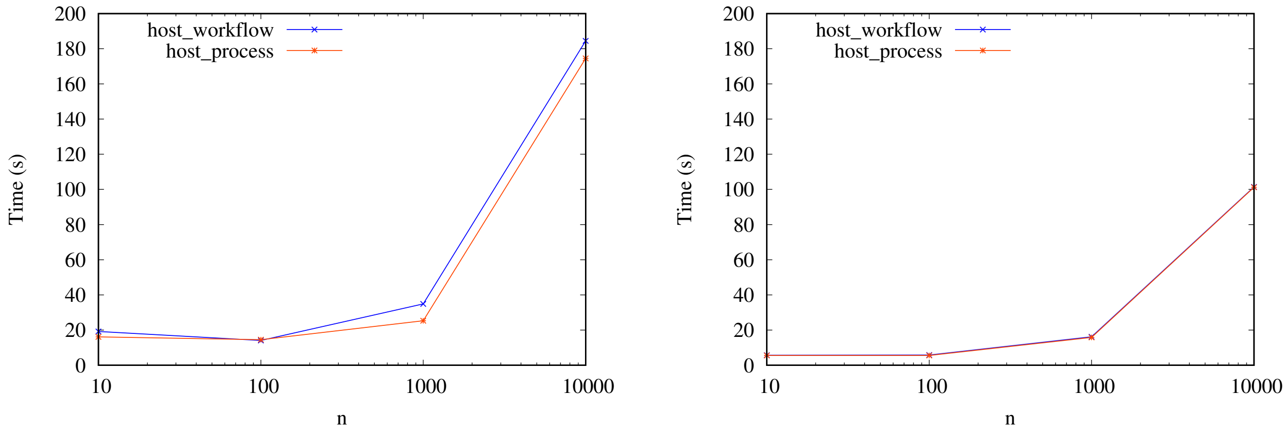

(a) Nextflow results.

(b) DeBasher results.

Figure S42: Time cost of Debasher and Nextflow when executing an increasing number of tasks using the one- and the two-step programs, called `host_process` and `host_workflow`, respectively,

	**MAE**
**Nextflow (array size=1)**	2.12e-02
**Nextflow (array size=10)**	1.18e-03
**Nextflow (array size=100)**	6.00e-04
**Nextflow (array size=1000)**	5.63e-04
**Nextflow (array size=2000)**	4.96e-04
**Nextflow (array size=5000)**	6.25e-04
**DeBasher**	4.59e-04

Table S1: Deviation from the ideal distribution of tasks across cluster nodes measured in terms of MAE for Nextflow (with various array sizes) and DeBasher when executing `host_workflow` with 10K tasks.

13 Sample Bioinformatics Workflow

DeBasher was used to implement a variant calling bioinformatics pipeline. The pipeline was run over two whole genome sequencing cancer datasets: the melanoma dataset MELA-AU (dataset ID: EGAD00001003388; 183 individuals) and the esophagus dataset ESAD-UK (dataset ID: EGAD00001003580; 303 individuals). Both datasets are available at `https://ega-archive.org/`.

Figures S43 and S44 show a process and a dependency graph, respectively, for the executed pipeline.

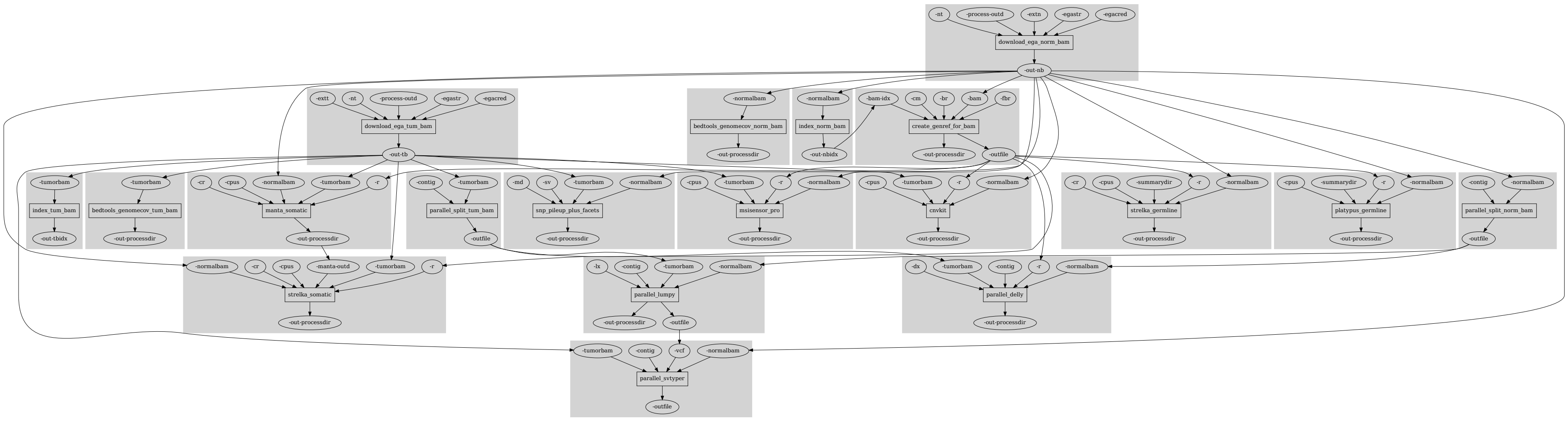

Figure S43: Process graph for a bioinformatics workflow implemented with DeBasher.

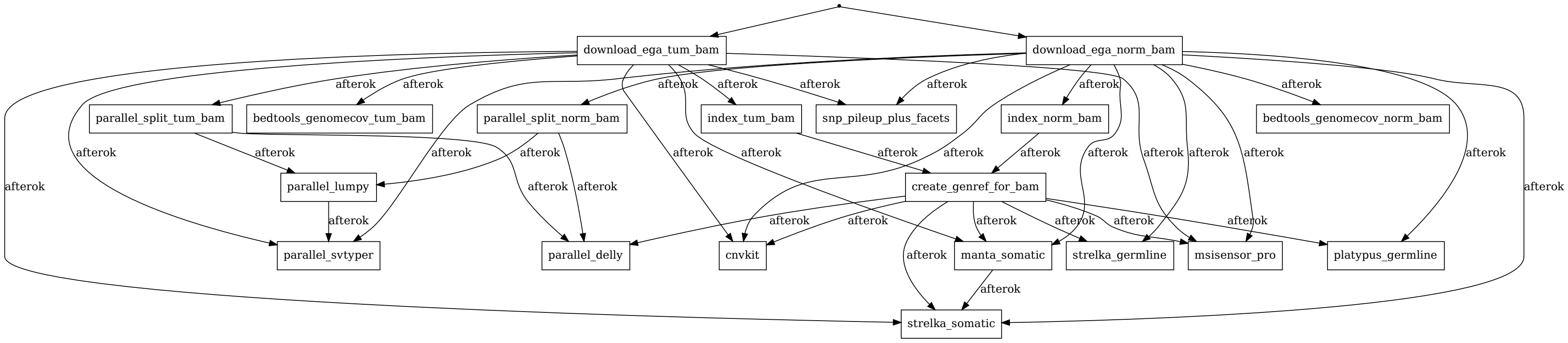

Figure S44: Dependency graph for a bioinformatics workflow implemented with DeBasher.

References

[1] Azza E. Ahmed, Joshua M. Allen, Tajesvi Bhat, Prakruthi Burra, Christina E. Fliege, Steven N. Hart, Jacob R. Heldenbrand, Matthew E. Hudson, Dave Deandre Istanto, Michael T. Kalmbach, Gregory D. Kapraun, Katherine I. Kendig, Matthew Charles Kendzior, Eric W. Klee, Nate Mattson, Christian A. Ross, Sami M. Sharif, Ramshankar Venkatakrishnan, Faisal M. Fadlelmola, and Liudmila S. Mainzer. Design considerations for workflow management systems use in production genomics research and the clinic. *Scientific Reports*, 11(1):21680, Nov 2021.

[2] Paolo Di Tommaso, Maria Chatzou, Evan W. Floden, Pablo Prieto Barja, Emilio Palumbo, and Cedric Notredame. Nextflow enables reproducible computational workflows. *Nature Biotechnology*, 35(4):316–319, Apr 2017.

[3] Johannes Köster and Sven Rahmann. Snakemake—a scalable bioinformatics workflow engine. *Bioinformatics*, 28(19):2520–2522, 08 2012.

[4] J.P. Morrison. *Flow-based Programming: A New Approach to Application Development*. J.P. Morrison Enterprises, second edition, 2011.

[5] Simon P. Sadedin, Bernard Pope, and Alicia Oshlack. Bpipe: a tool for running and managing bioinformatics pipelines. *Bioinformatics*, 28(11):1525–1526, 04 2012.

[6] John Vivian et al. Toil enables reproducible, open source, big biomedical data analyses. *Nature Biotechnology*, 35(4):314–316, Apr 2017.

[7] John Vivian, Arjun Arkal Rao, Frank Austin Nothaft, Christopher Ketchum, Joel Armstrong, Adam Novak, Jacob Pfeil, Jake Narkizian, Alden D. Deran, Audrey Musselman-Brown, Hannes Schmidt, Peter Amstutz, Brian Craft, Mary Goldman, Kate Rosenbloom, Melissa Cline, Brian O'Connor, Megan Hanna, Chet Birger, W. James Kent, David A. Patterson, Anthony D. Joseph, Jingchun Zhu, Sasha Zaranek, Gad Getz, David Haussler, and Benedict Paten. Toil enables reproducible, open source, big biomedical data analyses. *Nature Biotechnology*, 35(4):314–316, Apr 2017.
